# Repeated reunions and splits feature the highly dynamic evolution of 5S and 35S ribosomal RNA genes (rDNA) in the Asteraceae family

**DOI:** 10.1186/1471-2229-10-176

**Published:** 2010-08-16

**Authors:** Sònia Garcia, José L Panero, Jiri Siroky, Ales Kovarik

**Affiliations:** 1 Institut Botànic de Barcelona (CSIC-ICUB), Passeig del Migdia s/n, Parc de Montjuïc, 08038 Barcelona, Catalonia, Spain; 2 Institute of Biophysics, Academy of Sciences of the Czech Republic, Královopolská 135, CZ-612 65 Brno, Czech Republic; 3 Section of Integrative Biology, University of Texas, Austin TX 78712, USA

## Abstract

**Background:**

In flowering plants and animals the most common ribosomal RNA genes (rDNA) organisation is that in which 35S (encoding 18S-5.8S-26S rRNA) and 5S genes are physically separated occupying different chromosomal loci. However, recent observations established that both genes have been unified to a single 35S-5S unit in the genus *Artemisia *(Asteraceae), a genomic arrangement typical of primitive eukaryotes such as yeast, among others. Here we aim to reveal the origin, distribution and mechanisms leading to the linked organisation of rDNA in the Asteraceae by analysing unit structure (PCR, Southern blot, sequencing), gene copy number (quantitative PCR) and chromosomal position (FISH) of 5S and 35S rRNA genes in ~200 species representing the family diversity and other closely related groups.

**Results:**

Dominant linked rDNA genotype was found within three large groups in subfamily Asteroideae: tribe Anthemideae (93% of the studied cases), tribe Gnaphalieae (100%) and in the "Heliantheae alliance" (23%). The remaining five tribes of the Asteroideae displayed canonical non linked arrangement of rDNA, as did the other groups in the Asteraceae. Nevertheless, low copy linked genes were identified among several species that amplified unlinked units. The conserved position of functional 5S insertions downstream from the 26S gene suggests a unique, perhaps retrotransposon-mediated integration event at the base of subfamily Asteroideae. Further evolution likely involved divergence of 26S-5S intergenic spacers, amplification and homogenisation of units across the chromosomes and concomitant elimination of unlinked arrays. However, the opposite trend, from linked towards unlinked arrangement was also surmised in few species indicating possible reversibility of these processes.

**Conclusions:**

Our results indicate that nearly 25% of Asteraceae species may have evolved unusual linked arrangement of rRNA genes. Thus, in plants, fundamental changes in intrinsic structure of rDNA units, their copy number and chromosomal organisation may occur within relatively short evolutionary time. We hypothesize that the 5S gene integration within the 35S unit might have repeatedly occurred during plant evolution, and probably once in Asteraceae.

## Background

It is generally considered that in prokaryotes and in some species of early diverging eukaryote groups e.g. yeast, *Saccharomyces cerevisiae*, rRNA genes are organised in a single operon, clustered in tandem and transcribed by the same RNA polymerase. In eukaryotes, the 35S (encoding 18S-5.8S-26S rRNA) and 5S genes are transcribed by different polymerases, RNA polymerase I and III, respectively. Independent control of transcription probably enabled physical separation of both loci in chromosomes, an arrangement that is typical for most eukaryotic organisms. Nevertheless, it seems that there are several exceptions to this rule. For example, 5S genes linkage to other repetitive sequences including 35S, histone genes or the trans-spliced leader has been demonstrated [1]. These linked arrangements are found among diverse biological taxa including nematodes [2], fungi [3], crustaceans [4], slime moulds [5] or mosses [6,7], and they are believed to represent transition states between linked (prokaryotic) and unlinked (eukaryotic) arrangements. However, our recent observations in a group of angiosperms (genus *Artemisia*, Asteraceae) [8] clearly point to the possibility that linked arrangements might not be restricted to prokaryotes and primitive eukaryotes but may occur throughout the tree of life.

The Asteraceae, also named Compositae, is the largest family of the angiosperms in terms of numbers of species, with 1,620 genera and 23,600 species [9], constituting approximately 8-10% of all flowering plants. Although many trees and shrubs exist, a majority of species of Asteraceae are herbaceous, and they are easily recognizable by a suite of characters including fused anthers, a fruit with a single ovule, and a specialized inflorescence termed the capitulum. The family occurs in all continents of the world except Antarctica [9] and includes many edible, medicinal, noxious, invasive or endangered species. Estimates indicate that the Asteraceae originated in the mid Eocene (45-49 Mya) and that most tribal splits occurred during the Oligocene (28-36 Mya)[10]. The family has been the subject of intensive phylogenetic analyses using all kinds of molecular and morphological data [9,11] and it is currently considered to hold 12 major lineages [12], four of which (Asteroideae, Carduoideae, Cichorioideae and Mutisioideae) comprise 99% of its species diversity. Considering the size and importance of this family, however, relatively little has been published about rDNA position, organisation and structure in this group as a whole. In the past, the 5S and 35S genes have been mapped on chromosomes in some Asteraceae genera, e.g. *Tragopogon *[13], *Centaurea *[14], *Helianthus *[15], and *Hypochaeris *[16,17] among others, showing one or several separate loci of each, mostly in different chromosomes. In contrast, initial FISH mapping on *Artemisia *showed colocalised 35S and 5S signals [18-20]. Employing molecular methods we have recently demonstrated that several *Artemisia *species have evolved unusual ribosomal units resembling the arrangement in yeast: the 5S genes locate within the 26S-18S intergenic spacer and are transcribed from the opposite strand of the 35S rDNA operon [8]. In species displaying linked arrangement the homogenisation of linked rRNA genes most likely went to completion, since no unlinked genes were detected by FISH or Southern blot hybridisation. Taking into account the large size of the family it is not known how frequently such arrangement occurs, whether it is conserved in related species, its chromosomal dynamics and evolutionary success. With these issues in mind, we examined ribosomal DNA structure and organisation in selected representatives of the Asteraceae using molecular and cytogenetic methods. In addition, we searched for any nodes in extant Asteraceae phylogeny [21,22] that mark a switch in the organisation of rDNA.

## Methods

### Plant materials

Leaf or seed material for 199 populations of the different species studied was obtained either from wild populations, botanical gardens/herbaria worldwide, or through plants grown at the greenhouses of the Institute of Biophysics (Brno) and of the Botanical Institute of Barcelona, from seeds obtained by Index Seminum or purchased. Additional file 1 summarizes the provenance of all the taxa investigated.

### Southern blot hybridisation

Purified genomic DNAs and PCR products were digested with restriction enzymes and separated by gel electrophoresis on a 0.9% (w/v) agarose gel. After electrophoresis, the gels were alkali blotted onto Hybond-XL membranes (GE Healthcare, Little Chalfont, UK) and hybridised with ^32^P-labelled DNA probes (DekaLabel kit, MBI, Fermentas, Vilnius, Lithuania) as described in [8]. After washing under high-stringency conditions, the hybridisation bands were visualized with a PhosphorImager (Storm, Molecular Dynamics, Sunnyvale, CA), and the data were processed by ImageQuant software (Molecular Dynamics). The probes were a 220-bp PCR product derived from the 3' end of the 26S rRNA gene of tobacco [23] and a cloned ~120 bp of the 5S genic region, also from tobacco [24].

### Conventional PCR

**Figure 1:**
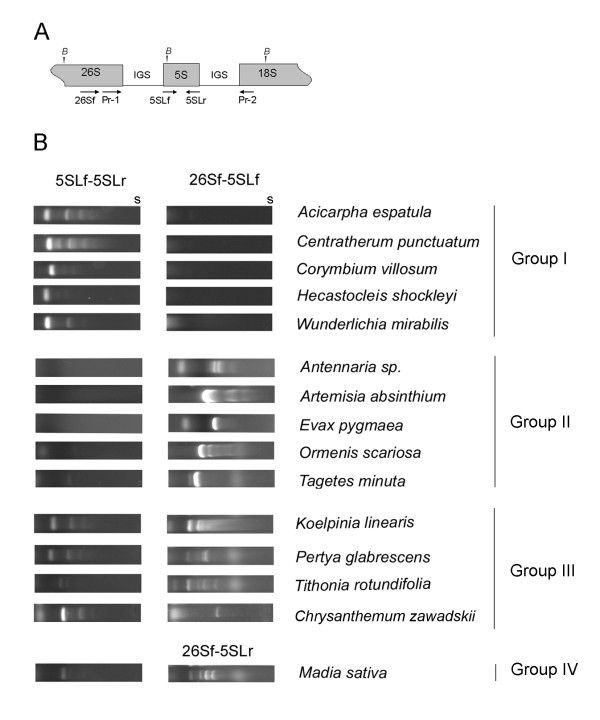
**PCR analysis of rDNA arrangements**. The upper panel (A) illustrates the strategy used for detection of mutual 26S and 5S positions. The position and orientation of primers are indicated by arrows. Grey boxes indicate coding sequences. *"B"*, cutting sites for *Bam*HI. The bottom panel (B) shows four profiles of PCR products obtained. "s", electrophoresis start.

Three PCR primers were used to amplify products assuming some linkage of 35S and 5S rRNA genes in either direct (head to tail) or inverted (tail to tail) orientations. The positions of the PCR primers are depicted in Figure 1. The primers were: 5SLf (5'-CCTGGGAATTCCTCGTGTT-3'), 5SLr (5'-TGCGTTAAAGCTTGTATGATCGCAT-3') from [24], and 26Sf (5'-GAATTCACCCAAGTGTTGGGAT-3'), originally called P1 in [23]. The PCR profile used for amplification was: initial denaturation at 94°C for 3 min followed by 35 cycles of 20 s at 94°C, 30 s at 57°C and 30 s at 72°C; and a final extension step at 72°C, 10 min.

### Real time PCR

Quantification of the rRNA gene copies was done using the SYBR Green PCR Master Mix (SAbiosciences, Fredrick, MD, USA) by the 7300 Real Time PCR System (Applied Biosystems/Ambion, Austin, TX, USA). The 26S-5S unit was amplified with the Pr-1 (5'-AGACGACTTAAATACGCGAC-3') and 5SLf primers; the control P2P gene was amplified with the forward primer 5'-CTGGATTTGCTGGTGATGAT-3' and the reverse primer 5'-CYCTCTTGGATTGAGCTT-3' [25] in the same PCR cycle to get an estimate of DNA concentration (initial denaturation at 94°C for 7 min followed by 40 cycles of 20 s at 94°C, 20 s at 56°C and 30 s at 72°C; and a final extension step at 72°C, 10 min). The SYBR Green I fluorescence was monitored consecutively after the extension step. A standard curve was made of series dilutions of the pAA_IGS-12 plasmid containing a cloned IGS from *A. absinthium *[GenBank accession number: EU649669]. This curve was then used to estimate amounts of rDNA in each of the samples based on the cross point values. The number of genes were calculated according to the formula: N = m/M × C × 1/10 where m is the amount of rDNA in ng; M, amount of genomic DNA input in ng; C, genome size in kb; 1/10 is a constant related to average size of rDNA unit (10 kb). The genome size data were taken from [26-28] and Plant DNA C-values Database (http://data.kew.org/cvalues/[29]).

### Cloning experiments

To isolate intergenic spacer sequences we used Pr-1 and Pr-2 (5'-GGCTTAATCTTTGAGACAA-3') primers [30]. The DyNAzyme™II DNA polymerase (Finnzymes, Espoo, Finland), was used in these PCRs; each reaction contained ~100 ng of genomic DNA. Following PCR, the products were gel-purified and the isolated fragments (QIAquick PCR purification kit, Hilden, Germany) were cloned into the pDrive vector (QIAGEN PCR Cloning Kit (Qiagen). Several positive clones were recovered from each transformation reaction and analysed for the lengths of insert by gel electrophoresis. Only clones containing inserts > 3 kb in size were further considered. The full length IGS constructs were obtained from *Tagetes patula *(pTPa_IGS, two clones), *Matricaria matricarioides *(pMMa_IGS, three clones), *Gnaphalium luteoalbum *(pGLu_IGS, three clones), *Helichrysum cymosum *(pHCy_IGS, two clones), *Madia sativa *(pMSa_IGS, two clones), *Elachanthemum intricatum *(pEIn_IGS, four clones) and *Helianthus annuus *(pHAn_IGS, two clones). The clones were sequenced both from the side of 26S and 18S genes (Eurofins, MWG Operon, Eberberg, Germany). The newly identified sequences were submitted to the EMBL/GenBank under the accession numbers [HM160523-29].

### Phylogenetic analysis

Sequences were assembled with the help of Bioedit software [31], manually edited and aligned. Phylogenetic relationships between clones were inferred from phylograms constructed based on the neighbor-joining (NJ) algorithm with the setting of the Kimura two-parameter substitution model. The statistical support for branching was obtained with a bootstrap analysis: 500 repetitions were carried out using the PHYLOWIN program [32]. The distances between individual clones were calculated with the assistance of DnaSP 4.0 software [33].

### Fluorescent in situ hybridisation

The fresh root tips were pretreated with an aqueous solution of colchicine 0.05% at room temperature, for 2.5 - 4 h and fixed in 3:1 (v/v) ethanol: acetic acid. Protoplasts were obtained using cellulolytic enzymes, dropped onto microscope slides, frozen and desiccated using liquid nitrogen and 70% ethanol. Before FISH, the slides were pre-treated with 50 μg mL^-1 ^RNaseA for 1 h at 37°C in humid chamber. After washing three times in 2× SSC (2× standard saline citrate + 0.1% (w/v) sodium dodecyl sulfate), slides were dehydrated in an ethanol series and air-dried. Remnants of cytoplasm were removed with pepsin treatment (10 μg mL^-1 ^in 10 mM HCl, 4 min room temperature). The slides were then washed, dehydrated in ethanol and fixed for 10 min in 3.7% formaldehyde in 1× PBS, washed three times in 2× SSC, dehydrated again and air dried. The hybridisation mix contained 50 ng μL^-1 ^of Cy3-labelled (GE Healthcare, Chalfont, St Giles, UK) 5S probe, a 116 bp-long insert of the cloned tobacco 5S rRNA gene [24], and 20 ng μL^-1 ^of 35S rDNA probe (a 2.5 kb fragment of 26S rRNA gene from tomato labelled with Spectrum Green, Abbott Molecular, IL, USA). The FISH hybridisation mixture (20 μL per slide) consisted of labelled DNA probes, 4 μL of a 50% solution of dextran sulphate, 10 μL formamide, 0.5 μL TE buffer and 2 μL 20 × SSC, and it was denatured at 75°C for 15 min and immediately cooled on ice. Slides were denatured in a thermocycler using a flat plate: 5 min at 75°C, 2 min at 65°C, 2 min at 55°C, 2 min at 45°C, and transferred into a pre-warmed moist chamber and put into an incubator. After overnight hybridisation at 37°C, the slides were washed with 2× SSC, 0.1× SSC (high stringency), at 42°C for 10 min each followed by washes with 2× SSC, 4× SSC + 0.1% Tween 20 at room temperature. Slides were rinsed in PBS and mounted in Vectashield (Vector Laboratories, Burlinghame, CA, USA) containing DAPI (1 μg/ml^-1^). FISH signals were observed using an Olympus AX 70 fluorescent microscope equipped with a digital camera. Images were analysed and processed using ISIS software (MetaSystems, Altlussheim, Germany).

## Results

### Species collections

We aimed to sample representative members of all subfamilies and most tribes within the Asteraceae (according to the recent phylogenetic revisions, see [22,34]) In decreasing order, the largest groups within this family are: subfamily Asteroideae, 17,000 species (73% of the family) > subfamily Cichorioideae (12.4%) > subfamily Carduoideae (11%). Out of the selected 199 accessions, 72% belong to subfamily Asteroideae, 9% to Cichorioideae and 7% to Carduoideae. The remaining 12% of the sample is composed by the other 9 subfamilies that complete the Asteraceae and by a representation of the sister families Calyceraceae and Goodeniaceae, the closely related Campanulaceae and Menyanthaceae, and the Solanaceae as a control as done in [10]. We consider that our sample selection is roughly reflecting distribution and relative abundance of species among the subfamilies and tribes. Multiple individuals per species were analysed and where possible different populations were scored. The complete list of taxa included in this study and details of specimens are given in Additional file 1.

### Genomic organisation of rDNA analysed by PCR

The PCR primers (Figure 1A) were used to amplify 5S genes with different genomic arrangements: (i) linked to the 26S gene (either in forward or reverse orientation, 26Sf-5Sf and 26Sf-5Sr), or (ii) long tandems of genes linked to its own via intergenic spacer (5Sf-5Sr) (Figure 1A). In total 199 DNA samples from 182 species were analysed with both sets of primers. Examples of amplification profiles are shown in Figure 1B and results summarized in Additional file 1. The DNA samples can be categorized into 4 groups according to PCR profiles:

1. DNAs producing amplification products with the 5SLf-5SLr primers but not with the 26Sf-5S primers (both combinations). These species were categorized as Group I species. Multiple regularly spaced bands in the 5S-5S profiles correspond to amplification of oligomeric units in tandems (mono-, di-, tri-, etc.). Thus Group I profile is consistent with a canonical separate unlinked tandem arrangement of 5S rRNA genes.

2. The DNA samples yielding strong 26Sf-5SLf PCR products and no 5SLf-5SLr products were categorized as Group II species. The patterns were interpreted as dominant linked 35S-5S arrangement in which the 5S genes are in an inverse orientation with respect to the direction of the 26S gene transcription.

3. There were cases of amplification products obtained with both 5SLf-5SLr and 26Sf-5SLf primer sets. This situation (Group III) might correspond to the presence of both linked and unlinked genes in the same genome, albeit in different proportions, as shown further.

4. Finally, few species such as *Madia sativa *(or *Mutisia speciosa *and also *Tithonia rotundifolia*, not shown) yielded amplification products with 26Sf-5SLr primer sets instead of the 26Sf-5SLf combination, that did not produce a product in these species), possibly indicating a direct orientation of the 26S-5S linkage at least in some units. These species (Group IV) also yielded positive PCR signals with the 5SLf-5SLr primer combination.

### Genomic organisation of rDNA analysed by Southern blot hybridisation

**Figure 2:**
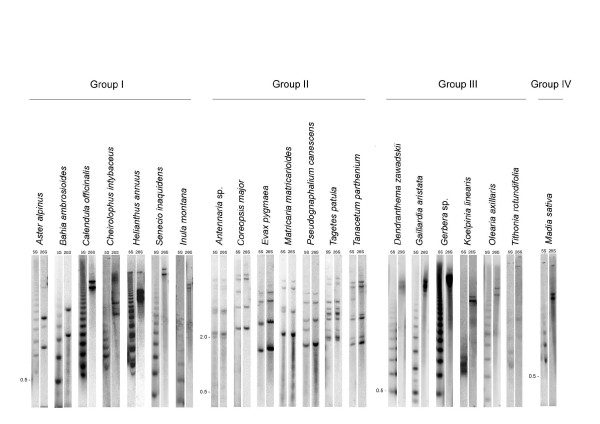
**Southern blot hybridisation analysis of rDNA arrangements**. The genomic DNAs representative species from each Group (I-IV) were digested with *Bam*HI and hybridised on blots with the respective DNA probes. The images were cut and arranged into panels, each representing individual species hybridised with the 5S and 26S probes. The sizes of monomeric units of tandemly arranged 5S repeats (Groups I, III and IV) were ~0.5 kb; the size of a major 26S-5S band in Group II species was 2-3 kb.

In order to validate and quantify the results of PCR analysis we carried out Southern blot hybridisation. We digested genomic DNA with *Bam*HI restriction enzyme, which has conserved recognition sites within both 5S and 26S genes. The blots were hybridised subsequently with the 26S, 5S and IGS probes. Examples of hybridisation profiles are shown in Figure 2 and results for all the species presented in Additional file 1.

Basically, two hybridisation profiles could be distinguished:

1. The 5S probe hybridisation bands having no overlap with the 26S hybridisation signals. These ladder profiles are consistent with long tandems of unlinked 5S genes. The 26S probe usually hybridised to several high molecular weight bands indicating the presence multiple IGS families lacking 5S insertions. Contrast to the 5S profile the 26S hybridisation did not generate a regular ladder of fragments which is explained by the presence of multiple *Bam*HI sites within the 35S unit (Figure 1A). These 5S and 26S hybridisation patterns were observed with all Group I, III and IV DNAs after the digestion with *Bam*HI. Patterns do not support the presence of long arrays composed of linked units in Group III and IV species despite positive PCR signals with the 26S-5S primer sets.

2. The Group II plants showed extensive cohybridisation of 26S and 5S probes to the same restriction fragments, consistent with dominant linked arrangement of both genes in the genome. The sizes of major fragments (2.5-3.0 kb) differed slightly between the species probably reflecting sequence polymorphism in the spacer between the 26S and 5S genes (confirmed by sequencing - see further below). In *Antennaria*, *Tagetes *and *Tanacetum *the major fraction (2.5-3.0 kb) migrated as doublets or triplets, indicating the presence of multiple gene families. On the other hand *Coreopsis*, *Evax*, *Matricaria *and *Pseudognaphalium*, had single bands in this region showing a single dominant family. The minor higher molecular weight fragments (> 5 kb) resulted most likely from inhibition of enzyme digestion due to methylation or mutation. Few 5S-hybridising fragments (e.g. in *Coreopsis*, *Matricaria*, *Pseudognaphalium *and *Tanacetum*) did not hybridise with the 26S probe while they did hybridise to the IGS probe from *Artemisia *(not shown).

### Quantitative PCR

**Figure 3:**
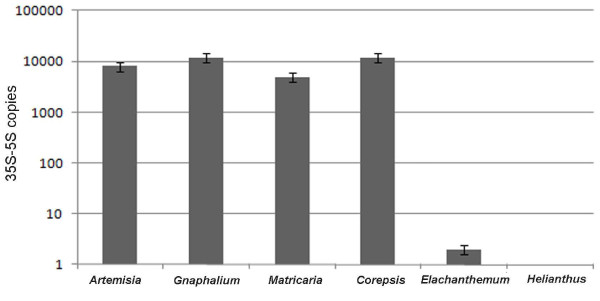
**Quantification of the linked rRNA genes copy number by real time PCR**. The Pr1-5SLf primer set (Figure 1A) was used to amplify inversely positioned 26S-5S genes. The graph shows mean values obtained from three independent experiments. Example of the amplification plot for *Artemisia *and *Elachanthemum *is shown in Additional file 2.

To determine the approximate copy number of linked rDNA units we carried out quantitative PCR. Since the sequences are amplified exponentially we expected that the high copy genes would need less cycles to amplify (low cross point values) than the low copy genes (high cross point values). With this supposition we amplified linked units using the Pr1-5SLf primers (Figure 1A). In accord with this, the cross point intervals for species with linked organisation (*Artemisia, Coreopsis*, *Gnaphalium *and *Matricaria*) were typically within 8-9 cycles using 100 ng DNA of template. In species with mostly unlinked arrangement the cross point intervals appeared after 25-28 cycles (*Elachanthemum intricanum*) or did not occur at all (*Helianthus annus*). Examples of amplification curves are shown in Additional file 2. These results revealed that there were approximately 5-10 thousand copies of linked 35S-5S units per Group II genome (Figure 3) whereas the Group I genomes had linked units in low copies (< 10) or they occurred below the detection limit. We also estimated the copy number of 26S genes using the specific primers. In this case, amplification plots were similar in all DNA samples irrespective of linked or unlinked genomic organisation (not shown).

### Structure of the 26S-18S intergenic spacers

**Figure 4:**
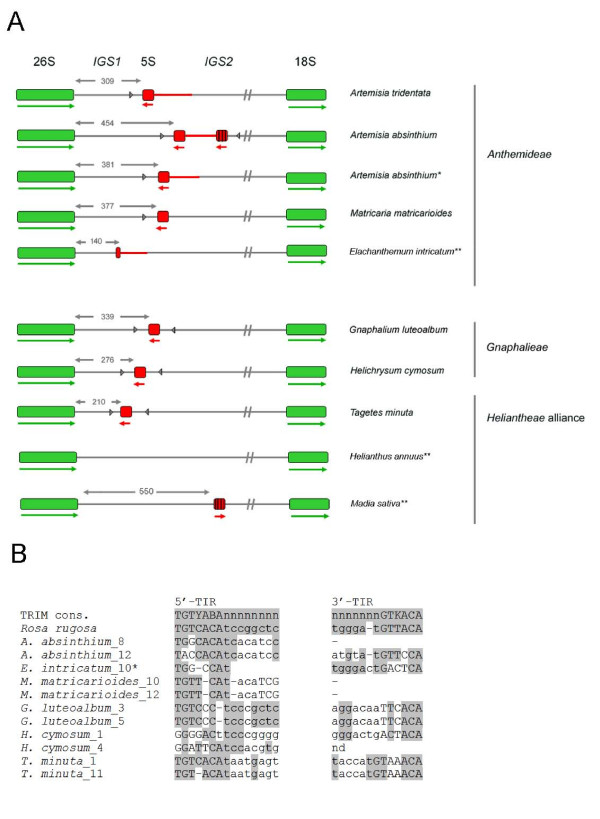
**The position and organisation of 5S insertions within the 26S-18S intergenic spacer**. (A). The 26S, 18S and 5S genic regions are in green and red boxes, respectively. Mutated or incomplete 5S copies are indicated by vertical strips. The sizes of IGS1 are depicted with thin lines above the units. Direction of transcription is illustrated by thick arrows below the genes. Arrowheads indicate sequences homologous to terminal inverted repeats (TIR) of Cassandra elements, aligned in (B). (*) the IGS type with a single 5S gene is the most abundant in *A. absinthium*; (**) species with typical unlinked arrangement of units.

To determine the structure of intergenic spacer and linked 5S genes we amplified the sequences between 26S and 18S genes. The templates included genomic DNA from species bearing linked (Group II species) and unlinked arrangement of rRNA genes (Groups I, III and IV). In each species between 2 to 3 clones were sequenced from the side of the 26S and 18S genes. The organisation of the IGS is schematically drawn in Figure 4A. With the exception of *Madia sativa*, the inverse orientation of 5S insertion(s) was highly conserved between the species. The longest IGS1 appeared to be present in *Artemisia absinthium *(454 bp) while the shortest was found in *Tagetes minuta *(210 bp). These assumptions are also consistent with the variable size of major *Bam*HI fragments on the Southern blots (Figure 2). It is likely that evolution of IGS1 was accompanied by numerous deletion and insertion events.

**Figure 5:**
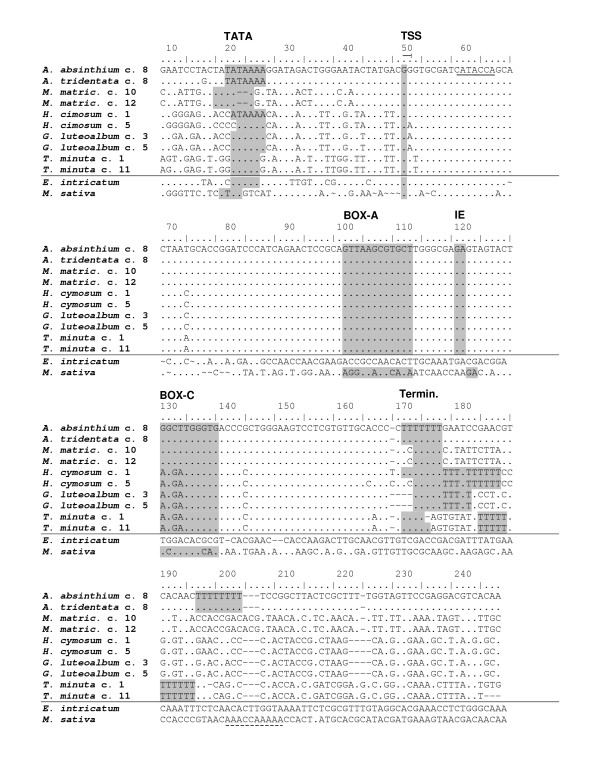
**Alignment of 5S genic regions and flanking sequences**. Conserved regulatory motifs essential for the polymerase III transcription [51] are highlighted. The insertions with complete and incomplete 5S copies are separated by a horizontal line. Note the highly conserved genic regions are well separated from the highly diverged 5' and 3' sequence ends. The 3' flanking sequences are only partially aligned because of the divergence. The numbers after the species name in titles indicate clones. TATA - TATA box; TSS - transcription start site; IE - internal regulatory element; Termin. - transcription terminator. The bottom strands (with respect to the 26S gene transcription) are shown except for *Madia sativa*. Underlined solid line - a motif resembling reverse transcriptase primer binding site (methionine tRNA). Underlined dashed line - a motif resembling a polyadenylation site.

Surprisingly, sensitive pairwise comparisons (5S versus IGS) revealed partial homologies to 5S genes in species that have evolved unlinked arrangement of units. For example, *Elachanthemum *IGS carries a 5S remnant in close proximity to the 26S gene. *Madia sativa *IGS contained a highly mutated (52% homology) 5S sequence ~ 350 bp downstream from the 26S gene (Figure 4A), a peculiar feature of this presumed 5S insertion is its direct orientation with respect to the 26S gene. In *Elachanthemum *and *Madia *both regulatory regions (A and C boxes) were mutated or deleted (Figure 5), suggesting that these 5S insertions are not transcribed and probably represent non-functional pseudogenes. There were no apparent 3' terminators while there was a motif resembling cellular polyadenylation signal in the sequence of *Madia*. No 5S-like sequences were found in the IGS of *Helianthus*. The 5S remnants within the IGS could potentially explain the presence of 26Sf-5SLf (r) PCR products in Group III and IV species.

Since 5S-homologous sequences have been found among the SINE3 retrotransposons in eukaryotic organisms [35], including plants [36], we searched the 5S flanking sequences for distinctive motifs typical of retroelements such as short repeats, long terminal repeats (LTR), transcription priming sites and duplications of 18S and 26S genes. All IGS analysed contained motifs resembling consensus sequences of terminal inverted repeats (TIR) of Cassandra retroelements (Figure 4B), which carry independently transcribed 5S rRNA genes [36]. Their position was variable within <200 bp upstream or downstream from the 5S genic region (Figure 4A). In some cases homology extended to the LTRs. Duplications of a putative integration site as well as domains typical from autonomous retrotransposons (e.g. reverse transcriptase) were, however, not found.

The aligned 5S sequences comprising both genic and flanking regions are shown in Figure 5. In species that homogenised the 35S-5S units (Group II plants) the linked 5S copies contained all regulatory elements to execute transcription by polymerase III: the A and C boxes were found at conserved positions. While box A was invariant there was slight sequence variation in box C. The transcription starts (TSS) at G is preceded by a highly conserved C at -1. The TATA sequence appeared to be fairly conserved located about 24-30 bp upstream from the transcription start site. Most species had two oligo dT terminators in the 3' flanking region while *Helichrysum cymosum *sequence bears a single 16-17 bp-long T track immediately downstream from the genic region. In short, the species with homogenised linked arrangements bear apparently functional 5S genes in the 26S-18S intergenic spacer(s).

The sequence divergences along individual regions of units are given in Additional file 3. As expected, both 26S and 5S genic parts were highly conserved while the inter- and intra genic spacers were less conserved. There was a 10 fold difference in divergence levels between genic and non-genic regions. Thus, the 5S insertions evolve in concert with linked 18S-5.8S-26S genes.

### Phylogenetic relationships between rRNA gene families

**Figure 6:**
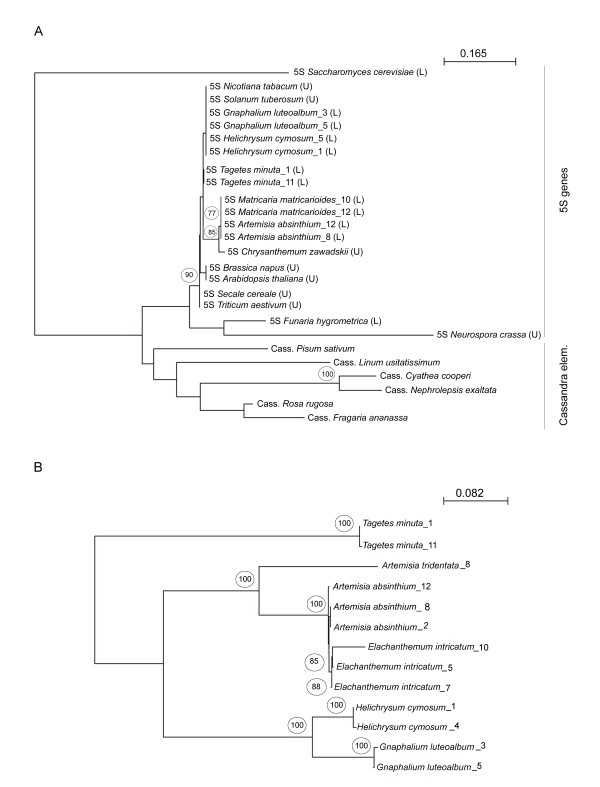
**Phylogenetic analysis of the 5S genic (A) and intergenic spacer 1 (IGS1) sequences (B)**. The arrangement of rRNA genes is depicted by U (unlinked) and L (linked). The phylogenetic positions are based on the nearest neighbor analysis of multiple alignments. Accession numbers of taxa are given in Additional file 4. The "5S" and "Cass." symbols before the taxon name indicate either 5S genic or Cassandra sequences. Numbers above branches indicate bootstrap percentages >70. The *Saccharomyces cereviseae *5S gene was used as outgroup in (A). The IGS1 cladogram is an unrooted tree. Scale bars indicate the number of base substitutions per site.

Using neighbour joining analysis we constructed a tree comprising several representative 5S sequences bearing linked and unlinked arrangement of rDNA (Figure 6A). We also included 5S sequences located within the LTRs of recently discovered Cassandra retroelements in other angiosperm species [36]. Both linked and unlinked 5S genes from angiosperms clustered into the same branch. However, there was poor resolution inside the clades, indicating none or little phylogenetic signal at this taxonomic level. We conclude that switches in the arrangement of 5S genes probably did not influence their homogeneity, structural and functional features. *Funaria hygrometrica*, a moss bearing linked rRNA genes [6] appears in a separate branch from the angiosperm sequences, as does the mold *Neurospora crassa*. In contrast with the genic sequences, the 5S within the LTR of Cassandra transposons form separate highly diversified branches consistent with the previously constructed tree [36].

The phyogenetic tree constructed from the IGS1 sequences is shown in Figure 6B. The topology of major clades is reminiscent to that previously published [21,37]. Well supported branches at the genus levels are of note.

### Fluorescent in situ hybridisation

**Figure 7:**
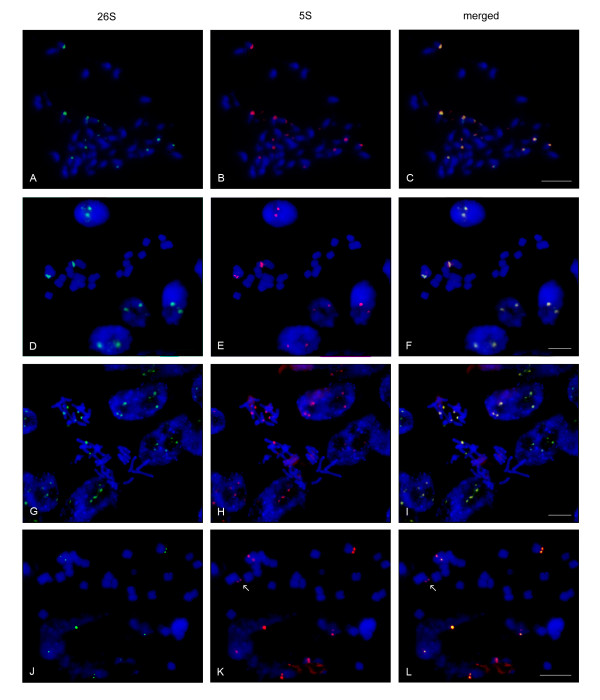
**Fluorescent *in situ *hybridisation of metaphase chromosomes and interphase nuclei of species that evolved linked arrangement of rRNA genes: (A-C) *Tagetes patula *(2n = 48); (D-F) *Helichrysum bracteatum *(2n = 22); (G-I) *Tripleurospermum maritimum *(2n = 36), (J-L) *Coreopsis major *(2n = 24)**. The 35S and 5S loci are labelled in green and red, respectively. Arrows indicate a possible solo 5S locus in *Coreopsis major*.

**Figure 8:**
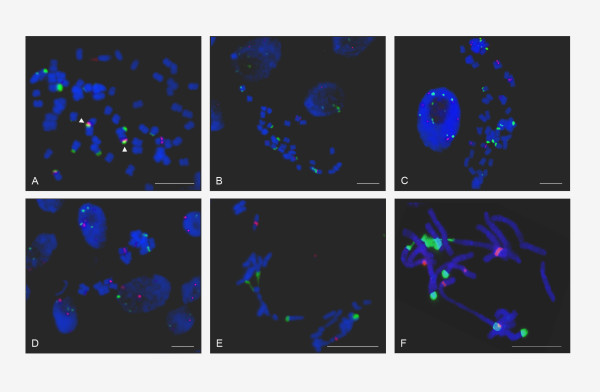
**Fluorescent *in situ *hybridisation of metaphase chromosomes and interphase nuclei of species that evolved unlinked arrangement of rRNA genes**. The individual slides show merged 26S and 5S signals: (A) *Dahlia pinnatta *(2n = 64), (B) *Helianthus annuus *(2n = 34); (C) *Chrysanthemum zawadskii *(2n = 54), (D) *Aster alpinus *(2n = 18); (E) *Calendula officinalis *(2n = 28); (F) *Tragopogon mirus *(2n = 24). Note that the 26S loci are mostly terminal whereas the 5S sites are mostly interstitial. Arrowheads in (A) indicate juxtaposition of 35S and 5S arrays without physical linkage of units.

To reveal chromosomal positions of rDNA loci and the degree of repeat homogenisation we conducted FISH assays in selected representatives of the Asteroideae (group where most linked arrangements have been found) and in one Cichorioideae (Figures 7, 8). We carried out bicolour labelling of metaphase chromosomes and interphase nuclei using the 26S (green fluorescence) and 5S (red flourescence) rDNA probes. In Figure 7, the representative Group II species were analysed. The 26S, 5S hybridisations are shown in the left and middle columns, respectively; the merged signals are arranged the right column. Most signals were subterminal. In *Tagetes patula *(Tageteae, from "Heliantheae alliance"), *Helichrysum cymosum *(Gnaphalieae), *Tripleurospermum maritimum *(Anthemideae) and *Coreopsis major *(Coreopsidae, from the "Heliantheae alliance") both probes cohybridised and no major signals of isolated 35S or 5S rRNA genes were visible. An exception to the rule could be a faint 5S locus in *Coreopsis *that failed to hybridise with the 26S probe. However, we do not know whether this was caused by weak 26S hybridisation or a true absence of 26S genes in the locus.

Figure 8 shows result of FISH analysis in representative Group I species with separate rDNA arrangement. *Dahlia pinnata *(Coreopsidae, from "Heliantheae alliance"), *Helianthus annus *(Heliantheae, also from "Heliantheae alliance"), *Chrysanthemum zawadskii *(Anthemideae), *Aster alpinus *(Astereae), *Calendula officinalis *(Calenduleae), and *Tragopogon mirus *(tribe Cichorieae, from subfamily Cichorioideae) present typical unlinked rDNA arrangement with 5S located interstitially and the 35S loci being terminal. One rDNA locus in *Dahlia pinnata *showed partial overlap of both 26S and 5S probes.

A summary of FISH data for all species is presented in Table 1. Given that the sensitivity limit of FISH is about 10-50 kb [12], equalling to tandems of twenty to hundred 5S or one to five 35S rRNA genes, we can conclude that there are probably no long tandems of unlinked 5S or 35S copies in most species of Group II genomes. Conversely, in Group I genomes there are no significant arrays bearing linked units.

**Table 1:** Summary of cytogenetic analysis by rDNA-FISH.

*Species^1^*	*Tribe*	*2n*	*Ploidy level*	*Linked^2 ^35S-5S loci*	*Unlinked^2 ^5S loci*	*Unlinked^2 ^35S loci*
*Artemisia absinthium*	Anthemideae	18	2	2	0	0
*Artemisia tridentata*	Anthemideae	18	2	3	0	0
*Aster alpinus*	Astereae	18	2	0	2	1
*Calendula officinalis*	Calenduleae	28	nd^3^	0	1	2
*Dahlia pinnata*	Heliantheae alliance	64	4	0	2	5
*Chrysanthemum zawadskii*	Anthemideae	54	6	0	3	5
*Coreopsis major*	Heliantheae alliance	24	2	3	(1)^4^	0
*Helianthus annus*	Heliantheae alliance	34	2	0	3	2
*Helichrysum bracteatum*	Gnaphalieae	22	nd^3^	2	0	0
*Tagetes patula*	Heliantheae alliance	48	4	7	0	0
*Tripleurospermum maritimum*	Anthemideae	36	4	4	0	0

^1 ^for *A. Absinthium *and *A. tridentata *the data were taken from [8]; the remaining species were analysed in this work (Figures 7, 8).

^2 ^number of sites per haploid set

^3 ^not determined

^4 ^minute 5S locus apparently devoid of 35S genes

## Discussion

### Two contrasting modes of rDNA organisation in Asteraceae

**Figure 9:**
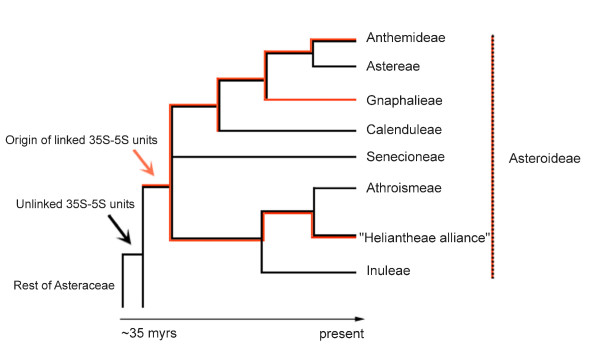
**Phylogenetic distribution of rDNA arrangement in the subfamily Asteroideae**. All groups showing homogenised linked arrangement are present. Tree topology is according to the published phylogeny [21]. Red and black lines indicate evolutionary trajectories of linked and unlinked units, respectively.

In this work, we investigated genomic and chromosomal organisation of 35S and 5S rRNA genes in ~200 plant species from the 12 subfamilies currently recognized in Asteraceae [21,22] and from other closely related families as control. We observed frequent physical linkages of both genes in the subfamily Asteroideae, whereas the remaining 12 subfamilies and the other families studied had a separate organisation. Thus, linked arrangement of rDNA arose independently in the largest subfamily. However, there are differences between these tribes: the linked arrangement was frequently found in Anthemideae, Gnaphalieae and in "Heliantheae alliance" but it is apparently not present in tribes Astereae, Calenduleae, Senecioneae, Inuleae and Athroismeae (Figure 9). Tribe Anthemideae comprises ~1,800 species (~7.6% of the family), "Heliantheae alliance" ~5,700 species (~24% of the family) and tribe Gnaphalieae around 1,200 species (~5% of the family). The high incidence of linked arrangement in the latter tribe (100% according to the studied species) is also supported by the recent observation of 35S-5S linkage among species of genus *Achyrocline *[38]. In contrast to Gnaphalieae a variable number of species with unlinked rRNA genes has been found in Anthemideae (7%) and "Heliantheae alliance" (77%). For example, *Chrysanthemum zawadskii *or *Elachanthemum intricatum *of tribe Anthemideae have unlinked organisation whereas most members of the tribe studied (93% cases) show linked arrangement of rDNAs. In "Heliantheae alliance" there is probably even more variation: the commercial accessions of sunflower, *Helianthus annuus *and *Dahlia pinnata *present an unlinked arrangement (Figure 8), whereas *Tagetes patula *and *Coreopsis major *(Figure 7) show linked rRNA genes. *Coreopsis *and *Dahlia *are members of tribe Coreopsideae, nevertheless, they show contrasting patterns of rDNA arrangements. Finally, there may also exist dispersed low copy 5S genes (either complete or remnants) linked to 26S genes (and perhaps to other repeats) as evidenced by PCR signals (Group III and IV species). These loci probably escape FISH or Southern blot detection limits (Figure 2 and Additional file 1).

The assorted situation here resembles that of primitive eukaryotes (yeast or other fungi) in which 5S genes display either linked arrangement with the other rDNAs, unlinked tandems, or even a dispersed organisation [3]. The results obtained in this study are significant since they show variation in rDNA arrangements even between species assumed to diverge relatively recently, such as the mentioned case of the very closely related genera *Elachanthemum *and *Artemisia*. Based on molecular clock dating, it appears that the family is not older than 50 Myrs [39] and species within most tribes probably diverged recently [40]. Together, linkage cannot be considered as an exclusive characteristic of the family, subfamily or tribe since significant rearrangements of rDNA probably occur at lower taxonomic levels such as the genus.

### Mechanisms of rDNA rearrangements

**Figure 10:**
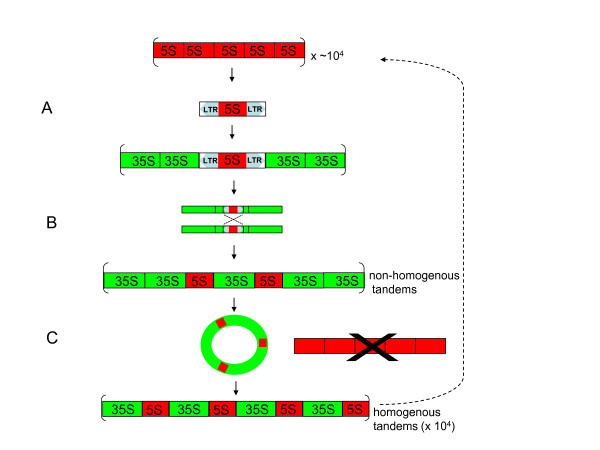
**Hypothetical model illustrating a cascade of events accompanying 5S rDNA evolution in Asteraceae**. (A) Mobilisation of 5S genes by a retrotransposition event and their integration within the 35S unit; (B) short (< 100 units) tandems of 35S-5S repeats generated by intralocus non-homologous recombination; inactivation of retroelement activity by mutation and/or epigenetic silencing. (C) amplification and spreading of 35S-5S arrays across the chromosomes mediated by mechanisms such rolling circle replication and reintegration of covalently linked circles. Loss of arrays of unlinked units. Green rectangles - units of 35S genes, red rectangles - units of 5S genes. Dotted line depicts the reversion process from linked to unlinked genotype. For the simplicity, proportions of gene sizes are not in scale.

Based on the widespread occurrence of separate organisation of rRNA genes in angiosperms, it is plausible to assume (although not proved) that the most primitive organisation was the unlinked from which the linked arrangement eventually evolved. Our data show that ~25% of Asteraceae species homogenised several thousands of linked units to completion without evidence for long arrays of unlinked loci, indicating that at some stage the unlinked arrays have been replaced by the linked through concerted evolution. Several hypotheses involving DNA recombination and RNA mediated transposition have been previously proposed to explain appearance of linked rRNA genes in fungi and nematodes [1]. We have adapted these hypotheses to explain this astonishing efficiency of rDNA unit replacement. Our model (Figure 10) considers a cascade of genetic events that may be fast or slow, unidirectional or reversible.

1. The initial phase probably involved a retrotransposition event that inserted a 5S gene(s) into the IGS (Figure 10A). This assumption is supported by several retroelement features found in the IGS of many of the studied species: (i) imperfect short 4-7 bp terminal inverted repeats, resembling those of non-autonomous TRIM retroelements called Cassandra (carrying 5S-like sequences, [36]), were found in close proximity to the 5S insertions (Figure 4B). In some species (e.g. *G. luteoalbum*), the homology apparently included several additional base pairs of Cassandra LTR; (ii) motifs resembling potential primer binding sites (PBS) for reverse transcription (minus strand) occur not only in the IGS of some species [8] but, interestingly, also within the genic region (close to the 5'end) pointing to the retrotransposon origin of 5S genes, in general.

It also has to be mentioned that there are features that distinguish the 5S IGS insertions from Cassandra retroelements. First, the 5S insertions in IGS contain all structural features of functional 5S genes at least in species that homogenised linked units to completion. On the other hand the 5S-LTRs of the sequenced Cassandra clones are mutated and unlikely to encode functional RNA molecules. This is supported by the phylogenetic analysis that failed to distinguish between linked and unlinked cellular copies of 5S but clearly separated these from Cassandra sequences (Figure 6A). The second distinction is that while termination signals follow immediately after the 5S coding region in the genic 5S, absence of terminators in Cassandra sequences extends polymerization to LTR by >400 nt [36]. It is unlikely that molecules of this size would be accurately processed and packed into ribosomes. Clearly, Cassandra elements, on their own, could not serve as 5S vehicles that carried functional 5S genes into the IGS. However, while there are thousands of similar copies in plant genomes we cannot exclude the possibility that there may be a yet undiscovered Cassandra locus bearing a functional 5S gene. Alternatively, a recombination event could have occurred between a retroelement and 5S tandems in a putative Asteroideae progenitor giving rise to a novel element. The presence of 5S-5S spacer sequences and duplication of 5S insertions in some IGS clones (Figure 4) could support the hypothesis.

While the divergence of 5S flanking sequences (Figures 5 and Additional file 3) point to multiple integration events, an alternative interpretation is also possible since the part of IGS downstream from the 26S gene is under low selective constraint (the lowest within the 35S unit), and residing sequences (tandem repeats and minisatellites) may evolve and change rapidly [41,42]. In this scenario, an ancestral 35S-5S unit may have undergone a series of deletion and mutation events in intergenic spacers, perhaps inactivating transposon activity, while selection has apparently acted to maintain coding regions. The fact that functional 5S genes are linked to the non coding 35S strand in all cases observed is also in line with a single origin of 35S-5S repeats.

2. Concerted evolution. FISH and quantitative PCR experiments showed that in many genomes concerted evolution has homogenised thousands of linked units indicating that insertion of a putative 5S-carrying retroelement into IGS does not prevent unit amplification. This may be consistent with situation in tomato [41], *Drosophila *[43] and nodding onion [44] in which units bearing retroelement insertions were observed in high copy. Classical theoretical models explained homogenisation of tandem repeats by multiple non-homologous recombination steps [[43] and references herein]. Computer simulations imply that homogenisation time of a tandemly arranged sequence is a quadratic function of copy numbers [45]. It follows that 10^4 ^copies of a typical rDNA array homogenise within 10^8 ^years. However, the Asteroideae subfamily is probably not older than 26-39 million yrs [9,10]. Many of its members with contrasting rDNA arrangements (e.g. *Tagetes *v. *Helianthus *or the previously mentioned *Elachanthemum *v. *Artemisia*) probably diverged more recently. In addition, some species, e.g. *Tagetes patula*, carry arrays of homogenised 35S-5S genes distributed across many chromosomes suggesting extensive interlocus recombination and/or recurrent polyploidy events. Thus, while the model based solely on non-homologous recombination between chromatids seems to work with yeast (mainly due to the lower number of rDNA copies, less than 200) and may elucidate the initial phase of 35S-5S repeat expansion (Figure 10B), an alternative model is needed to explain such rapid turnover of thousands of rRNA genes and their spreading across the chromosomes. Several mechanisms including rolling cycle replication and reintegration of covalently linked circles [46,47] have been recently proposed to mediate homogenisation of satellite repeats in allopolyploids [48] and may be possibly applied to the rDNA switches as well (Figure 10C).

3. It is intriguing that we were unable to identify a species bearing equivalent proportion of linked and unlinked tandems, since FISH studies confirmed homogenisation across all rDNA loci (Figure 7 and Table 1). Either mixed genotypes are counterselected (intermediate stages, Figure 10B) may not be evolutionarily successful or the turnover (birth and extinction) of rDNA arrays in genomes is extremely fast so intermediate genotypes are short-lived and escape detection. The best evidence for the latter hypothesis is the organisation of rDNA in *Elachanthemum intricatum*, an Old World Anthemideae species. In this species, the functional rRNA genes are unlinked [8]. However, a non-functional 5S remnant was found in the 26S-18S IGS. Its strand orientation, proximity to the 26S gene and flanking sequences were similar to functional 5S-IGS copies in the closely related *Artemisia *species (Figure 4A). These data suggest that a putative *Elachanthemum *progenitor did have linked arrangement of units that have become eventually lost. Hypothetically, a large deletion of locus or a chromosome loss could have promoted reamplification of unlinked 5S arrays and reversion of the unlinked rDNA genotype (Figure 10C). It will be interesting to analyse Asteraceae genomes (which are now largely unexplored) by deep sequencing in order to reveal intermediate stages of rDNA rearrangements, to map potentially active retroelement loci that mobilised 5S genes, and to determine the degree of tandem arrays homogeneity, in general.

### The rDNA arrangement as a potential phylogenic tool

The intra- and interspecies divergence rates (Additional file 3) for 26S and 5S genes were comparable (low) confirming concerted evolution of the 35S-5S units and corroborating studies in yeasts [49]. In contrast, the sequence between the 26S and 5S genes (termed IGS1) is not conserved and displays significant length and sequence polymorphisms (Figure 4). The cladogram based on these sequences showed highly supported branches (Figure 6B) and the tree topology presented good congruence with other phylogenetic reconstructions using different markers, as mentioned previously. The divergence of IGS1 was significantly higher than that of ITS1 (Additional file 3): between closely related species e.g. *Artemisia tridentata *and *A. absinthium*, the divergence of ITS1 was ~4% (10 substitutions per 250 bp) whereas for IGS1 the divergence was ~7% (35 substitutions per 488 bp). Thus IGS1 could be used as a suitable, alternative marker to infer phylogenetic relationships in taxonomically difficult taxa, e.g. Asteroideae. Besides, on the basis of the finding of linked arrangement in Anthemideae, Gnaphalieae and "Heliantheae alliance", maybe phylogenetic relationships between these groups should be reassessed, as these groups could be more closely related than currently considered [9,21]. However, present nuclear and chloroplastic molecular phylogenies [[11] and references therein] confirm that, though related, these groups do not form a clade, but that Heliantheae alliance's sister group are tribes Athroismeae and Inuleae, and that Anthemideae are sister to Astereae (Figure 9). So in fact, our findings could reflect the lability of rDNA arrangements in these species.

The rearrangement of intrinsic locus structure in which thousands of copies are lost and replaced with new copies certainly represents a drastic genomic change with potential phylogenetic signal. Though a dual 5S and 35S FISH appears to be a reliable method to determine organisation of rDNA loci on chromosomes, it is somewhat impractical for large scale sampling as ours. In addition, the signals overlap may not necessarily mean physical linkage but rather juxtaposition of both arrays as demonstrated in the case of *Dahlia pinnata *(Figure 8). The simple PCR methodology described in Figure 1, based on primers amplifying 26S-5S and 5S-5S sequences, may represent a feasible approach.

## Conclusion

Family Asteraceae nowadays occupy all kinds of habitats, from hot deserts to high mountain areas. Just in genus *Artemisia *there may have been 17-22 direct and 2-4 reverse migrations from Asia to America [50]. We have shown that the unusual linked organisation of 35S-5S rDNA units occurs in three large groups of Asteraceae family comprising species-rich genera including abundant and economically important plants. The units were homogenised within the loci and across the chromosomes to several thousands of copies, with concomitant loss of the independent 5S and 35S tandems. Given that these tribes represent a large proportion of the species richness of the family we estimate that nearly a quarter of Asteraceae species could have evolved a linked arrangement of rRNA genes. We can therefore envisage that the linked rDNA arrangement in plants may be far more common than previously thought. Uncovering the adaptive advantages (if any) of a linked rDNA arrangement is the next step in the understanding of the dynamic evolution of rRNA genes in plants.

## Authors' contributions

SG and AK designed the study and wrote the paper. SG carried out most molecular biology and cytogenetic experiments; AK carried out bioinformatic studies and drafted the paper, JS participated in the cytogenetic part. JP was involved in the phylogenetic characterisation of species and DNA sampling. All authors read and approved the final manuscript.

## Supplementary Material

Additional file 1 **List of species studied, together with an indication of their origin and results of rDNA arrangement analysis**. In columns "26Sf-5SLf" and "5SLf-5SLr": (+) indicates presence and (-) absence of PCR product with these primer combinations. In general, a product with the 26Sf-5SLf primers column points to linkage of 26S and 5S rRNA genes whereas (+) in the "5SLf-5SLr" column suggests a tandem arrangement of 5S rRNA genes, hence, unlinked rRNA genes in most cases; (vw) very weak, (w) weak, (s) strong and (vs) very strong, refer to the intensity of PCR products, and (f) fragments, indicates presence of fragments of different sizes in electrophoresis gels. Column "*Bam*HI" shows results of Southern blot obtained after digestion with this enzyme and hybridisation with 26S and 5S rDNA probes: (L) linkage of both rRNA genes, i. e. both probes hybridising with the same restriction fragments (Group II species), (NL) non-linkage of rRNA genes, i. e. both probes hybridise with different restriction fragments (Group I, III and IV species). Examples of PCR and Southern blot hybridisation gels are shown in Figures 1 and 2. Click here for file

Additional file 2 **Example of amplification plot**. The data were obtained from real time amplification reaction in the presence of SYBR green fluorescence dye for high and low copy. After the run the products were analysed by gel electrophoresis (right margin). Click here for file

Additional file 3 **Comparison of divergences of genic and intergenic regions**. The distances (Pi) between individual clones were calculated with the assistance of DnaSP 4.0 software [33]. The sequences were obtained in this study or downloaded from the EMBL/GenBank database. The data sets comprised rDNA sequences from *Artemisia *(2 species), *Tagetes*, *Matricaria*, *Helichrysum *and *Elachanthemum*. Click here for file

Additional file 4 **Accession number of DNA sequences used for construction of phylograms**. Click here for file
