# Supplementary material for: Repeated reunions and splits feature the highly dynamic evolution of 5S and 35S ribosomal RNA genes (rDNA) in the Asteraceae family
Source: BMC Plant Biol. 2010 Aug 16;10:176. doi: 10.1186/1471-2229-10-176 (PMC3095306; doi:10.1186/1471-2229-10-176)
Supplement: Additional file 1 — List of species studied, together with an indication of their origin and results of rDNA arrangement analysis. In columns "26Sf-5SLf" and "5SLf-5SLr": (+) indicates presence and (-) absence of PCR product with these primer combinations. In general, a product with the 26Sf-5SLf primers column points to linkage of 26S and 5S rRNA genes whereas (+) in the "5SLf-5SLr" column suggests a tandem arrangement of 5S rRNA genes, hence, unlinked rRNA genes in most cases; (vw) very weak, (w) weak, (s) strong and (vs) very strong, refer to the intensity of PCR products, and (f) fragments, indicates presence of fragments of different sizes in electrophoresis gels. Column "BamHI" shows results of Southern blot obtained after digestion with this enzyme and hybridisation with 26S and 5S rDNA probes: (L) linkage of both rRNA genes, i. e. both probes hybridising with the same restriction fragments (Group II species), (NL) non-linkage of rRNA genes, i. e. both probes hybridise with different restriction fragments (Group I, III and IV species). Examples of PCR and Southern blot hybridisation gels are shown in Figures 1 and 2. [file 1471-2229-10-176-S1.PDF]

Additional file1. List of species studied, together with an indication of their origin and results of rDNA arrangement analysis. In columns “26Sf-5SLf” and “5SLf-5SLr”: (+) indicates presence and (-) absence of PCR product with these primer combinations. In general, a product with the 26Sf-5SLf primers column points to linkage of 26S and 5S rRNA genes whereas (+) in the “5SLf-5SLr” column suggests a tandem arrangement of 5S rRNA genes, hence, unlinked rRNA genes in most cases; (vw) very weak, (w) weak, (s) strong and (vs) very strong, refer to the intensity of PCR products, and (f) fragments, indicates presence of fragments of different sizes in electrophoresis gels. Column “*Bam*HI” shows results of Southern blot obtained after digestion with this enzyme and hybridisation with 26S and 5S rDNA probes: (L) linkage of both rRNA genes, i. e. both probes hybridising with the same restriction fragments (Group II species), (NL) non-linkage of rRNA genes, i. e. both probes hybridise with different restriction fragments (Group I, III and IV species). Examples of PCR and Southern blot hybridisation gels are shown in Figs. 1 and 2.

SUBFAMILY	TRIBE	SPECIES	26SF-5SLF	5SLF-5SLR	BamHI	ORIGIN
Asteroideae	Anthemideae	*Ajania achilleoides*	+ vs, f	+ f	L	Kazakhstan. A. A. Ivaschenko et al. S-2098, 2000. BCN 25624.
		Ajania fastigiata	+	-	L	People's Republic of China: Xingian-Uigur, Kashgar. M. Petrov, 1959. LE.
		Ajania fruticulosa	+	-	L	Mongolia: Southern Gobi. Sh. Dariimaa, Sh. Tsooj, J. Vallès and E. Yatamsuren, 2004 BCN.
		Anthemis mauritanica	+	-	L	Spain: Catalonia, Barcelona. Botanical Garden of Barcelona, vii.2008. BCN.
		Artemisia absinthium	+	+ w	L	Spain: Catalonia, Setcases. S. Garcia and C. Ibarria ix. 2008. BCN.
		Artemisia absinthium	+	-	L	Spain: Catalonia, Pyrenees. T. Garnatje, S. Siljak-Yakovlev and J. Vallès. vii. 2008. BCN.
		Artemisia annua	+	-	L	Spain. M.Torrell and J. Vallès 1997. BCN 12486.
		Artemisia arbuscula ssp. *arbuscula*	+	-	L	USA: Nevada, Lander Co. S. Garcia SC46, E.D. McArthur, S.C. Sanderson and J. Vallès. 2008. BCN.
		Artemisia arbuscula ssp. *longicaulis*	+	-	L	USA: Nevada, Washoe Co. S. Garcia, SC49, E. D. McArthur, S. C. Sanderson and J. Vallès. 2008. BCN.
		Artemisia bigelovii	+	-	L	USA: Cocconino Co., Arizona. Padre Canyon. E. D. McArthur 3051. 2006. BCN.
		Artemisia crithmifolia	+	-	L	The Netherlands: Haarlem. S. Garcia SC5 and C. Ibarria. iii.2008. BCN.
		Artemisia desertii	+	+ f	L	Iran: Semnan. BCN 13322.
		Artemisia dracunculus	+	+	L	Spain: Catalonia, Pyrenees. M. Torrell and J. Vallès 1997. BCN 13328.
		Artemisia frigida	+	-	L	Russia: Tüva, Kyzyl. J. Vallès. BCN 16421.
		Artemisia herba-alba ssp. *herba-alba*	+	-	L	Spain: Catalonia, Segrià. M. Torrell and J. Vallès 1996. BCN.
		Artemisia nova	+	-	L	USA: Utah, Millard County,Tunnel Spring, desert experimental range. 2005. E. D. McArthur 2876.
		Artemisia pygmaea	+	-	L	USA: Utah, Juab County, Yuba Dam road. 2005. E. D. McArthur 2870.
		Artemisia tridentata ssp. *tridentata*	+	-	L	USA: Utah, Juab County, Salt Creek Canyon. 2005. E. D. McArthur 2871.
		Artemisia tridentata ssp. *vaseyana*	+ f	+ w	L	USA: Utah, Juab County, Salt Creek Canyon. 2005. E. D. McArthur 2872.
		Artemisia tridentata ssp. *wyomingensis*	+ vs, f	-	L	USA: Utah, Millard County. S. Garcia SC39 E. D. McArthur, S. C. Sanderson and J. Vallès. 2008. BCN.
		Artemisia tridentata ssp. *wyomingensis*	+	-	L	USA: Utah, Uintah County. S. Garcia SC27 E. D. McArthur, S.C. Sanderson and J. Vallès. 2008. BCN.
		Artemisia tridentata ssp. *wyomingensis*	+ vs, f	+ vw	L	USA: Utah, Carbon County, Gordon Creek.2005. E. D. McArthur 2886.
		Artemisia tripartita ssp. *tripartita*	+	-	L	USA: Nevada, Clark County. Dubois Sheep Station. 2005. E.D. McArthur 2845. BCN.
		Artemisia vulgaris	+	-	L	Portugal: Vila Nova de Gaia. M. Torrell and J. Vallès 1997. BCN 15273.
		Athanasia trifurcata	+	-		Spain: Catalonia, Barcelona. Botanical Garden of Barcelona, vii.2008 BCN.
		Brachanthemum gobicum	+	-		Mongolia: Southern Gobi. Sh. Dariimaa, Sh. Tsooj, J. Vallès and E. Yatamsuren. 2004. BCN.
		Chrysanthemum arcticum ssp. *maekawanum*	+	+	NL	Japan: Sapporo, Sapporo Botanical Garden. IS 2003. BCN S-815.
		Chrysanthemum indicum var. *coreanum*	+ n	+ n	NL	Japan: Sapporo, Sapporo Botanical Garden. IS 2003. BCN S-809.
		Chrysanthemum indicum var. *indicum*	+ n-s	+ n	NL	Czech Republic: Moravia, Brno. Florist's shop. Garcia and Garnatje xi. 2007. BCN.
		Chrysanthemum maximowiczii	+	+	NL	Russia: Glazkovka. Vladivostok IS N70. BCN S-810.
		Chrysathemum zawadskii	+ n	+	NL	Japan: Sapporo, Sapporo Botanical Garden. IS 2003. BCN S-814.
		Elachanthemum intricatum	+ w	+ f		Mongolia: Dzhung Gobi aymak. Grubov et al. 1979. LE.
		Elachanthemum intricatum	+ w	+ f	NL	Mongolia: Southern Gobi. Sh. Dariimaa, Sh. Tsooj, J. Vallès and E. Yatamsuren. 2004. BCN.
		Eriocephalus africanus	+	-	L	Spain: Catalonia, Barcelona. Botanical Garden of Barcelona. S. Garcia and M. Veny. i.2007.
		Filifolium sibiricum	+	-		People's Republic of China: Ordos desert. Krasnovorob n. n. 1988. LE.
		Hyppolitia megacephala	+	-		Kazakhstan. A. A. Ivaschenko et al. S-2169 2000. BCN 11695.
		Kaschgaria brachanthemoides	+	-		Kazakhstan. A. A. Ivaschenko et al. S-2129 2000. BCN 11691.
		Lepidolopsis turkestanica	+	+		Kazakhstan. A. A. Ivaschenko et al. S-2209 2000. BCN 25629.

SUBFAMILY	TRIBE	SPECIES	26SF-5SLF	5SLF-5SLR	BamHI	ORIGIN
		Leuchanthemum maximum	+	+	L	Czech Republic: Bohemia, Dobříš. Nohel Garden. S. Garcia i.2009. BCN.
		Lonas annua	+ vw	+ 3f		Spain: Catalonia, Barcelona. Botanical Garden of Barcelona. ix.2007. BCN.
		Matricaria matricarioides	+	-	L	USA: Wyoming, Albany County. S. Garcia SC41 E. D. McArthur, S. C. Sanderson and J Vallès. ix.2008. BCN.
		Mausolea eriocarpa	+	-		Uzbekistan: Bukhara. Sandy desert, 10 km from Gazli. L. Kapustina et al S-2041. 3.xi.1999. BCN 11629.
		Neopallasia pectinata	+	-		People's Republic of China: Xingjan-Uigur. Southern slope of eastern Tien-Shan. Lunatov U-Fen s. n. 1958.
		Nipponanthemum nipponicum	+			Japan: Higashi-Hiroshima. K. Kondo. 2003. BCN S-811.
		Oncosiphon sabulosum	+	-		Republic of South Africa: Western Cape Province, Cape of Good Hope Nature Reserve. A. Romo R-14530, M. Galbany, S. Arrabal and M. Koekemoer ii.2007. BCN.
		Ormenis scariosa	+ f	+ w		Spain: Catalonia, Barcelona. Botanical Garden of Barcelona. S. Garcia and M. Veny. i.2007.
		Phaeostigma salicifolium	+	-		People's Republic of China: Tangut, Kansu. Southern Tepun Mountains. LE n. n.
		Picrothamnus desertorum	+	-		USA: Wyoming, Sweetwater County, Winton Road. 2004. E.D. McArthur 2403.
		Santolina chamaecyparissus	+	-	L	Spain: Catalonia, Barcelona. Botanical Garden of Barcelona. S. Garcia and M. Veny. i.2007.
		Spaheromeria ruthiae	+	-	L	USA: Utah, Washington County, Zion Natnl. Park, Refrigerator Canyon. 2003. E. D. McArthur 1775.
		Sphaeromeria argentea	+	-	L	USA: Wyoming, Sweetwater County, South of Chimney Rock. 2005. Goodrich and Atwood 22533.
		Sphaeromeria cana	+	-		USA: Nevada, Nye County. Broom Canyon. 2002. Goodrich, Smith and Tuhy 20075.
		Sphaeromeria capitata	+	-		USA: Colorado, Moffat County. S. Garcia SC40, E. D. McArthur, S. C. Sanderson and J. Vallès. ix.2008. BCN.
		Sphaeromeria diversifolia	+	-		USA: Utah, Utah County. Santaquin Canyon. E.D. McArthur and J. Vallès. 2000. BCF 49505.
		Sphaeromeria potentilloides	+	-		USA: Camas County, South of Hill City. 2004. E. D. McArthur 2425.
		Tanacetum ferulaceum	+	+	L	Spain: Catalonia, Barcelona. Botanical Garden of Barcelona. T. Garnatje and M. Veny. ix.2007. BC.
		Tanacetum vulgare	+ s-vs	+	L	Spain: Catalonia, Barcelona. Botanical Garden of Barcelona. S. Garcia. i.2007.
		Tripleurospermum maritimum	+	-	L	Iceland: Akureyri. Sandy banks near sea level. IS 2004. BCN.
		Turaniphytum eranthemum	+	-		Kazakhstan: Almatynskaya oblast. Ivaschenko et al. S-2143 2000. BCF 50684.
		Ursinia saxatilis	+	+ f		Republic of South Africa: Kwazulu-Natal Province, 25 km from Underberg. A. Romo R-14383, M. Galbany, S. Arrabal and M. Koekemoer. ii.2007. BCN.
	Astereae	*Aster alpinus*	-	+s	NL	Czech Republic: Bohemia, Dobříš. Nohel Garden. S. Garcia i.2009.
		Aster hymalaicus	-	+ s	NL	Czech Republic: Bohemia, Dobříš. Nohel Garden. S. Garcia i.2009.
		Chrysothamnus nauseosus	-	-		Germany: Frankfurt. Botanischer Gärten Dr. Johann Wofgang Goethe-Universität Frankfurt am Main. IS 2008.
		Conyza sumatrensis	+	-	NL	Spain: Catalonia, Barcelona. Botanical Garden of Barcelona. S. Garcia and M. Veny, iii.2007. BCN.
		Grindelia robusta	-	+ w		Spain: Catalonia, Barcelona. Botanical Garden of Barcelona. S. Garcia, S. Pyke and M. Veny. vii.2008.
		Grindelia squarrosa	+	+ f	NL	Germany: Bonn, Botanischer Gärten der Universität Bonn (Ref. xx-0-Bonn-6124). IS 2007.

SUBFAMILY	TRIBE	SPECIES	26SF-5SLF	5SLF-5SLR	BamHI	ORIGIN
		Haplopappus foliosus	-	+		Spain: Catalonia, Barcelona. Botanical Garden of Barcelona, ix.2007.
		Heterotheca subaxillaris	-	+ vw		Spain: Catalonia, Barcelona, Botanical Garden of Barcelona, ix.2007. BCN.
		Machaeranthera canescens	-	-		USA: Utah, Wasatch Co., Center Creek Canyon. S. Garcia SC31, E. D. McArthur, S. C. Sanderson, J. Vallès, ix.2008. BCN.
		Olearia axillaris	+ w	+ s	NL	Spain: Catalonia, Barcelona. Botanical Garden of Barcelona, iv.2008.
		Townsendia hookeri	-	+ f		USA: Washington, Graham, Mt Tahoma Nursery. J. L. Panero 2010-1. TEX.
	Bahieae (Heliantheae alliance)	*Bahia ambrosioides*	+ s, f	-	NL	Spain: Catalonia, Barcelona. Botanical Garden of Barcelona. v.2008. BC.
		Bahia dissecta	+w	-	NL	USA: Nevada, Clark County, Spring Mountains. S. Garcia SC16, E. D. McArthur, S. C. Sanderson and J. Vallès. ix.2008. BCN.
	Calenduleae	*Calendula officinalis*	-	+	NL	Czech Republic: Bohemia, Dobříš. Nohel Garden. S. Garcia i.2009.
		Calendula sp.	-	+ f	NL	Spain: Catalonia, Barcelona. Botanical Garden of Barcelona. S. Garcia. i.2007. BCN.
		Calendula trypterocarpa	-	+	NL	Spain: Catalonia, Barcelona. Botanical Garden of Barcelona. S. Garcia. i.2007. BCN.
		Chrysanthemoides monilifera	-	+ f		South Africa: Cape Town. Kirstenbosch Botanical Garden. Panero 2002-5. TEX.
		Dimorphoteca ecklonis	-	+	NL	Spain: Catalonia, Barcelona. Botanical Garden of Barcelona. S. Garcia and M. Veny. v.2007. BCN.
	Chaenactideae (Heliantheae alliance)	*Chaenactis douglasii*	+ w, f	+ w, f	NL	USA: Utah, Wasatch County, Strawberry Valley. S. Garcia SC34, E. D. McArthur, S. C. Sanderson and J. Vallès. ix.2008. BCN.
	Coreopsideae (Heilliantheae alliance)	*Coreopsis major*	+ f	-	L	Germany: Göttingen. Botanical Garden Göttingen. IS 2007. vii. 2008.
		Coreopsis pubescens	+	-	L	Czech Republic: Moravia, Brno. Brno Botanical Garden, S. Garcia xi.2007.
		Dahlia coccinea	+ f s	+	NL	Germany: Göttingen, Botanical Garden Göttingen. IS 2007.vii.2008.
		Dahlia pinnata	-	+	NL	Czech Republic: Dobříš, Nohel Garden. S. Garcia i.2009.
		Dahlia sp.	+ f	+	NL	Czech Republic: Bohemia, Prague. Prague Botanical Garden. C. Ibarria and S. Garcia xi.2007.
	Eupatorieae (Heliantheae alliance)	*Brickellia grandiflora*	+	-	NL	Switzerland: Saint Gallen. Botanical Garden Saint Gallen. IS 2007.
		Eupatorium cannabinum	+	+	NL	Spain: Catalonia, Vall d'Aran. E. Carrió, S. Garcia, K. Konowalik and J. Vallès x.2008.
		Eupatorium glandulosum	+ f	-	NL	Spain: Catalonia, Barcelona. Botanical Garden of Barcelona, S. Garcia and M. Veny. v.2008.
		Liatris ligustris				Germany: Bonn. Botanical Garden Bonn (Ref. xx-0-Bonn-9448). IS 2007.
	Gnaphalieae	*Antennaria* sp.	+	-	L	USA: Utah, Wasatch Co., Strawberry Valley. S. Garcia SC35, E. D. McArthur, S. C. Sanderson, J. Vallès, ix.2008. BCN.
		Antennaria sp.	+	-	L	USA: Utah, Wasatch Co., Strawberry Valley. S. Garcia SC37, E. D. McArthur, S. C. Sanderson, J. Vallès, ix.2008. BCN.
		Evax pygmaea	+	-	L	Switzerland: Saint Gallen. Botanical Garden Saint Gallen (Ref. xx-0-STGAL-15/1981-Freiland 2460) IS 2007.
		Gamochaeta pennsylvanica	+ vs	+ vw	L	USA: Texas. J. L. Panero 2003-27. TEX.
		Gnaphalium luteoalbum	+ vs	-	L	Spain: Catalonia, Barcelona. Botanical Garden of Barcelona. S. Garcia. i.2007. BCN.
		Gnaphalium supinum	+	-	L	Spain: Catalonia, Barcelona. Botanical Garden of Barcelona. S. Garcia. i.2007. BCN.
		Helichrysum bracteatum	+	-	L	Czech Republic: Bohemia, Dobříš. Nohel Garden. S. Garcia i.2009. BCN.
		Helichrysum cymosum	+ vs	+	L	Spain: Catalonia, Barcelona. Botanical Garden of Barcelona. S. Garcia and M. Veny. v.2007. BCN.
		Helichrysum retortum	+	-	L	Spain: Catalonia, Barcelona. Botanical Garden of Barcelona. S. Garcia. vii.2008. BCN.

SUBFAMILY	TRIBE	SPECIES	26SF-5SLF	5SLF-5SLR	BamHI	ORIGIN
		Pseudognaphalium canescens	+ f	-	L	USA: Utah, Washington Co., Zion National Park, Refrigerator Canyon. S. Garcia SC21, E. D. McArthur, S. C. Sanderson, J. Vallès, vii.2008. BCN.
	Helenieae (Heliantheae alliance)	*Gaillardia aristata*	+ sf n	+	NL	Germany: Berlin, Humboldt-Universität zu Berlin, Institut für Biologie Späth-Arboretum. IS 2007.
		Marshallia caespitosa	-	+ f		USA: Texas. J. L. Panero. 2008. TEX.
	Heliantheae (Heliantheae alliance)	*Berlandiera lyrata*	-	+	NL	France: Limoges, Jardin Botanique Ville de Limoges. IS 2007.
		Elaphandra paucipunctuata	-	+ w		Ecuador: Prov. El Oro. J. L. Panero 2008.
		Encelia farinosa	+	+ s	NL	Spain: Catalonia, Barcelona. Botanical Garden of Barcelona. T. Garnatje ix.2007. BC.
		Flourensia thurifera	-	+		Spain: Catalonia, Barcelona. Botanical Garden of Barcelona. T. Garnatje ix.2007. BC.
		Geraea canescens	-	+		USA: Arizona. J. L. Panero 2008.
		Helianthus annuus	+ vw	+ s,f	NL	Czech Republic: Bohemia, Dobříš. Nohel Garden. S. Garcia i.2009. BCN.
		Helianthus tuberosus	-	+ s,f	NL	Czech Republic: Moravia, Brno. Ruderal place. T. Garnatje xi.2007. BCN.
		Lagascea decipiens	-	+		Spain: Catalonia, Barcelona. Botanical Garden of Barcelona. T. Garnatje ix.2007. BC.
		Silphium sp.	+ s, f	+	NL	Czech Republic: Moravia, Brno. Ruderal place near the Institute of Biophysics. T. Garnatje x.2007. BC.
		Tithonia rotundifolia	+ s, f	+ f	NL	Germany: Bonn, Botanical Garden Bonn (Ref. xx-0-Bonn-21362). IS 2007.
		Venidium fastuosum	+ w	+	NL	Czech Republic: Bohemia, Dobříš. Nohel Garden. S. Garcia i.2009.
		Verbesina enceloides	+	+	NL	Switzerland: Saint Gallen. Botanical Garden Saint Gallen (Ref. xx-0-StGal-15/1986-Freiland) 1618). IS 2007.
		Verbesina helianthoides	+	+	NL	Germany: Bonn. Botanical Garden Bonn (Ref. xx-0-Bonn-9416). IS 2007.
		Viguiera laciniata	-	+ vw	NL	Spain: Catalonia, Barcelona. Botanical Garden of Barcelona. ix.2007. BCN.
	Inuleae	*Inula crithmoides*	-	+		Spain: Catalonia, Barcelona. Botanical Garden of Barcelona, vii.2008.
		Inula ensifolia	+ w, f	+ f	NL	Czech Republic: Bohemia, Prague. Prague Botanical Garden. C. Ibarria and S. Garcia xi.2007.
		Inula ensifolia	+ f	+ f	NL	Spain: Catalonia, Barcelona. Botanical Garden of Barcelona, ix.2007.
		Inula montana	-	-		Spain: Catalonia, Barcelona. Botanical Garden of Barcelona, vii.2008.
		Perralderia paui	-	+ w		Spain: Catalonia, Barcelona, Botanical Garden of Barcelona, ix.2007.
		Pulycaria disenterica	+ w, f	+ w		Spain: Catalonia, Barcelona. J. Vallès 2008.
		Schizogyne sericea	-	+ s f	NL	Spain: Catalonia, Barcelona, Botanical Garden of Barcelona, ix.2007.
		Telekia speciosa	+ w	+ w, f	NL	Italy: Udine. Orto botanico friulano "O. B. Didattico". IS 2007. BCN.
	Madieae (Heliantheae alliance)	*Arnica montana*	+ w	+	NL	Portugal: Oporto. Oporto University- Facuty of Sciences, Dep. of Botany, IS 2007. PO.
		*Madia sativa***	+ f	+	NL	France: Limoges. Jardin Botanique Ville de Limoges-France. IS 2007. vii.2008. Freiland 776.
	Millerieae (Heliantheae alliance)	*Guizotia abyssinica*	-	+ f	NL	France: Limoges. Jardin Botanique Ville de Limoges-France. IS 2007. vii.2008.
	Perityleae (Heliantheae alliance)	*Eutetras palmeri*	-	+ f		Mexico: Aguascalientes, J. L. Panero 7337, x.1997. TEX.
		Perityle emoryi	+ vw	+		Spain: Catalonia, Barcelona. Botanical Garden of Barcelona. T. Garnatje, ix.2007. BC.
	Plucheae	*Pluchea sericea*	+ vw	+	NL	Spain: Catalonia, Barcelona, Botanical Garden of Barcelona, ix.2007.

SUBFAMILY	TRIBE	SPECIES	26SF-5SLF	5SLF-5SLR	BamHI	ORIGIN
	Senecioneae	*Euryops annae*	+	+ f		Spain: Catalonia, Barcelona. Botanical Garden of Barcelona. S. Garcia. vii.2008. BCN.
		Tetradymia canescens		+		USA: Nevada, Lander Co. Austin summit. S Garcia SC47, E. D. McArthur, S. C. Sanderson, J. Vallès, ix.2008. BCN.
		Hertia cheirifolia	-	-		Switzerland: Saint Gallen. Botanical Garden Saint Gallen. IS 2007. vii.2008. BCN.
		Pericallis aurita	-	+ 2f s	NL	Spain: Catalonia, Barcelona. Botanical Garden of Barcelona. S. Garcia, M. Veny. i.2007. BCN.
		Psacalium paucicapitatum	+ f	+ s, f		Mexico: Oaxaca: J. L. Panero 2476. x.1991. TEX.
		Senecio inaquidens	-	+ f	NL	Austria: Salzburg. Botanical Garden Salzburg University IS 2007 (Ref. A, S, Hallein S, Gamp, industrial area, ruderal, calcareous soil, 440 msm 10/07).
		Senecio longifolius	+ vw	+ f	NL	Spain: Catalonia, Barcelona. Botanical Garden of Barcelona. S. Garcia. iii.2007. BCN.
		Senecio sp.	-	+ f	NL	Czech Republic: Moravia, Brno. T. Garnatje 2007. BCN.
	Tageteae (Heliantheae alliance)	*Porophyllum ruderale*	+	+ f	NL	France: Limoges. Jardin Botanique Ville de Limoges. IS 2007.
		Tagetes lucida	+	-	L	France: Limoges. Jardin Botanique Ville de Limoges. IS 2007.
		Tagetes minuta	+ vs, f	-	L	Germany: Bonn. Botanical Garden Bonn (Ref. YE-0-Bonn-21503). IS 2007.
		Tagetes patula	+ vs	+ f	L	Czech Republic: Moravia, Brno. Near the Institute of Biophysics, T. Garnatje, x.2007. BC.
		Tagetes patula	+ vs	+	L	Czech Republic: Bohemia, Dobříš. Nohel Garden. S. Garcia i.2009.
Barnadesioideae	Barnadeieae	*Barnadesia* sp.	-	-		Argentine: Jujiy, D. Gutiérrez, s.n., xi.2005.
		Chuquiraga sp.	+	-	NL	Argentine: Tucumán, Tafí del Valle, D. Gutiérrez, s.n., xi.2006.
		Schlechtendalia luzulaefolia	-	+	NL	Argentine: Buenos Aires. D. Gutierrez, s.n., iv.2009.
Carduoideae	Cardueae	*Arctium* sp.	-	+ s	NL	Czech Republic: Moravia, Brno. Bohunice kampus. Masaryk University, T. Garnatje, x.2007. BC.
		Atractylodes japonica	-	+ f		USA, Pennsylvania, Asiatica Nursery. J. L. Panero. TEX.
		Berardia subacaulis	-	+ w		France: Alpes Maritimes, Col de la Cayolle. T. Garnatje G27 and J. Luque. vi.2004. BC.
		Centaurea cyanus	+ vw	+	NL	Czech Republic: Bohemia, Prague. Prague Botanical Garden. IS 2008.
		Cheirolophus intybaceus	-	+ s	NL	Spain: Valencia, Bicorp. T. Garnatje and J. Vallès. i.2007. BCN.
		Cheirolophus intybaceus	-	+	NL	Spain: Alacant, Cap Negre. J. G. Segarra-Moragues. x.2008. BC.
		Cnicus benedictus	-	+		Switzerland: Saint Gallen. Botanical Garden Saint Gallen (Ref. xx-O-StGal-Freiland 8242) IS 2007.
		Echinops spinosus	-	+ f	NL	Spain: Catalonia, Barcelona. Botanical Garden of Barcelona, ix.2007. BC.
		Echinops strigosus	-	+	NL	Spain: Catalonia, Barcelona. Botanical Garden of Barcelona. T. Garnatje, vi.2008. BCN.
	Dicomeae	*Macledium zeyheri*	-	+		South Africa: Cape Town, Rhodes Drive, Newlands. Kirstenbosch Botanical Garden. S. Ortiz and J. L. Panero 2002-47. TEX.
	Oldenburgieae	*Oldenburgia grandis*	-	+		South Africa. Trinder-Smith s. n. US.
		Onopordum tauricum	-	-	NL	Switzerland: Saint Gallen. Botanical Garden Saint Gallen (Ref. xx-O-StGal-465/1988-Freiland 2532) IS 2007.
		Rhaponticum berardioides	+ s, f	+ s, f	NL	Morocco: Grand Atlas Central, Aït Bougmaze valley. O. Hidalgo and A. Romo R-12749. BC.
Cichorioideae	Arctotideae	*Dimondia margaretae*	-	+ s, f	NL	Spain: Catalonia, Barcelona. Botanical Garden of Barcelona. T. Garnatje, ix.2007.
		Heterolepis aliena	-	+		South Africa. Kirstenbosch National Botanical Garden. J. L. Panero 2002-35. TEX.
	Cichorieae	*Andryala chevalieri*	+ w	+	NL	Spain: Catalonia, Barcelona. Botanical Garden of Barcelona, M. *Veny*, ix.2007. BCN.
		Cichorium intybus	-	+ vw		Spain: Catalonia, Barcelona. Botanical Garden of Barcelona. T. Garnatje, ix.2007. BCN.

SUBFAMILY	TRIBE	SPECIES	26SF-5SLF	5SLF-5SLR	BamHI	ORIGIN
		Helminthoteca echioides	+ w	+ s, f		Portugal: Coimbra. Jardim Botânico Univ Coimbra-Portugal IS 2007. Ref. 449.
		Koelpinia linearis	+ f	+ f	NL	Germany: Rostock. Botanischer Garten Universität Rostock. IS 2007. Ref. F2896 2003 (160).
		Lapsana comunis	-	+ f		Italy: Udine. Orto botanico friulano "O. B. Didattico". Ref. F1388 (162) IS 2007.
		Launaea arborescens	-	+		Spain: Catalonia, Barcelona. Botanical Garden of Barcelona. S. Garcia. vii.2008. BCN.
		Rhagadiolus stellatus	-	+ f		Switzerland: Saint Gallen. Botanical Garden Saint Gallen (Ref. xx-0-STGAL-263/1988-Freiland 790) IS 2007.
		Scorzonera pseudopygmaea	-	+		Spain: Catalonia, Barcelona. Botanical Garden of Barcelona. S. Garcia. vii.2008. BCN.
		Sonchus leptocephalus	+ w	-	NL	Spain: Catalonia, Barcelona. Botanical Garden of Barcelona. T. Garnatje, ix.2007. BCN.
		Taraxacum sp.	-	+ s, f	NL	Czech Republic: Moravia, Brno. Near the Institute of Biophysics. T.Garnatje, x.2007.
		Tolpis succulenta	-	+		Spain: Catalonia, Barcelona. Botanical Garden of Barcelona. S. Garcia. vii.2008. BCN.
		Trommsdorfia maculata	-	-		Russia: Altai. South Siberian Botanical Garden of Altai State University. IS 2007.
		*Warionia saharae**	-	+ f	NL	Spain: Catalonia, Barcelona. Botanical Garden of Barcelona, ix.2007. BC.
	Gundelieae	*Gundelia tournefortii*	-	+		Iran. Al-Hosseini s. n. US.
	Liabeae	*Sinclairia palmeri*	-	+ f		Mexico: Durango, Km 92 of the road Durango-Mazatlan. J. L. Panero et al. 7457. TEX, IZTA, CIIDIR.
	Vernonieae	*Centratherum punctatum*	-	+ f		USA: cultivated. J. L. Panero 2002-53. TEX.
Corymbioideae	Corymbieae	*Corymbium villosum*	-	+	NL	South Africa: Cape Town. Purchased online at Silverhill seeds. J. L. Panero.
Gochnatioideae	Gochnatieae	*Cnicothamnus lorentzii*	-	+		Argentina. J.L. Panero 1934. TENN.
Gymnarrhenoideae	Gymnarrheneae	*Gymnarrhena micrantha*	-	+		Saudi Arabia. Mandeville 157. US.
Hecastocleidoideae	Hecastocleideae	*Hecastocleys shockleyi*	-	+		USA. J. L. Panero and B. S. Crozier 8157. TEX.
Mutisioideae	Mutisieae	*Adenocaulon bicolor*	-	+ s, f		USA. Twisselmann 7661. TEX.
		Gerbera × hybrida	+	+	NL	Czech Republic: Moravia, Brno, florist's shop, S. Garcia and T. Garnatje x.2007. BCN.
		Leibnitzia anandria	+ vw	+ w		USA: Pennsylvania, Warminster, Heronswood Nursery. J. L. Panero. TEX.
		Mutisia speciosa	-	- s, f		Brazil: Sao Paulo, J. Leiva s.n. TEX.
	Nassauvieae	*Lophopappus tarapacanus*	-	+ w		Chile: Arica, J. L. Panero and B. S. Crozier 8445. i.2004. CONC, TEX.
		Nassauvia digitata	-	+ s, f		Chile: Bio Bio. J. L. Panero and B. S. Crozier 8417. i.2004. CONC, TEX.
	Onoserideae	*Gypothamnium pinifolium*	-	+ s, f		Chile: Antofagasta. J. L. Panero and B. S. Crozier 8437. i.2004. CONC, TEX.
		Onoseris hastata	-	+ s, f		South America. Cultivated. Horn 1756. US.
Pertyoideae	Pertyeae	*Ainsliaea apiculata*	-	+		Japan: Ohtsuka s. n., Japan, no voucher (Panero and Funk 2008).
		Hyalis argentea	-	+		Argentina. Simon 657. US.
		Pertya glabrescens	+ s, f	+ s, f		Japan: Sapporo, Sapporo Botanical Garden. IS 2007. vii.2008.
Stifftioideae	Stifftieae	*Stifftia chrysanthemoides*	-	+ w		Brazil. Mikan, Serra 235. TEX.
Wunderlichioideae	Wunderlichieae	*Wunderlichia mirabilis*	-	+		Brazil. Riedel, Roque, Funk and Kim 466. US.

FAMILY	TRIBE	SPECIES	26SF-5SLF	5SLF-5SLR	BamHI	ORIGIN
**Sister families**						
Calyceraceae		*Acicarpha spathulata*	-	+ s, f		Brazil. Salgado 7660. TEX.
Goodeniaceae		*Lechenaultia formosa*	-	+ vs		Spain: Catalonia, Barcelona. Botanical Garden of Barcelona, vi.2008. S. Garcia, S. Pyke and M. Veny.
		Scaevola aemula	+ f	+ f	NL	Spain: Catalonia, Barcelona. Botanical Garden of Barcelona, vi.2008. S. Garcia, S. Pyke and M. Veny.
**Other families**						
Campanulaceae		*Campanula rotundifolia*	-	+ f	NL	Czech Republic: Bohemia, Prague. Prague Botanical Garden. IS 2008.
Menyanthaceae		*Menyanthes trifoliata*	-	+	NL	Spain: Catalonia, Vall d'Aran. E. Carrió, S. Garcia, K. Konowalik and J. Vallès, x.2008. BCN.
Solanaceae		*Nicotiana tabacum* "Vielbattriger"	-	+ f	NL	Slovakia: Bab. Cultivated. A. Kovarik. 2007.
