# Supplementary material for: Repeated reunions and splits feature the highly dynamic evolution of 5S and 35S ribosomal RNA genes (rDNA) in the Asteraceae family
Source: BMC Plant Biol. 2010 Aug 16;10:176. doi: 10.1186/1471-2229-10-176 (PMC3095306; doi:10.1186/1471-2229-10-176)
Supplement: Additional file 2 — Example of amplification plot. The data were obtained from real time amplification reaction in the presence of SYBR green fluorescence dye for high and low copy. After the run the products were analysed by gel electrophoresis (right margin). [file 1471-2229-10-176-S2.PDF]

Additional file 2. Real time PCR analysis of linked 26S-5S units in high copy number *Artemisia* and in low copy in *Elachanthemum.* Left: example of amplification plot. After the run the products were analysed by gel electrophoresis (right margin).

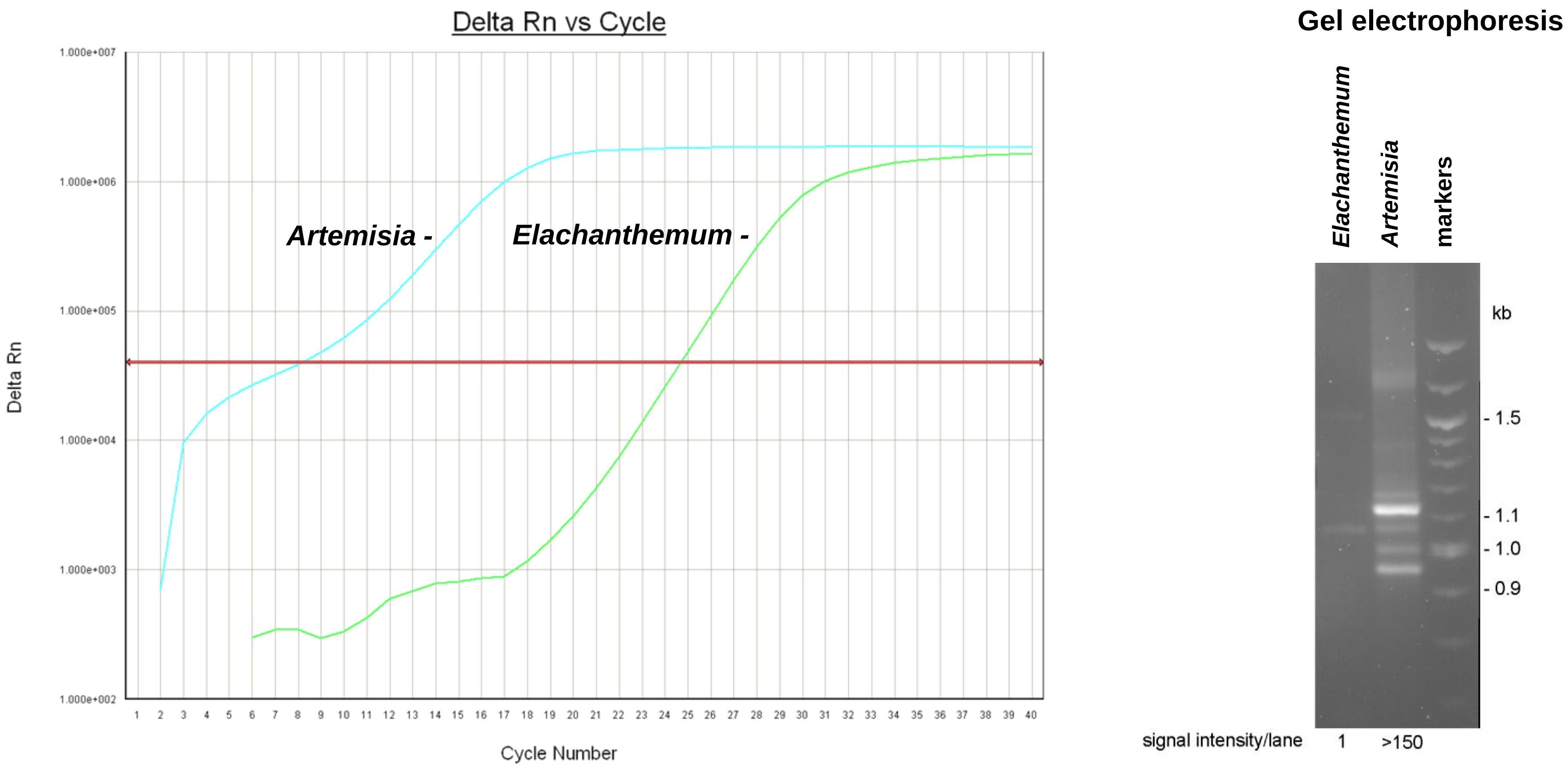

Result: there are ~ 8000 copies of linked genes in *Artemisia* and < 10 copies in *Elachanthemum*
