# Supplementary material for: Repeated reunions and splits feature the highly dynamic evolution of 5S and 35S ribosomal RNA genes (rDNA) in the Asteraceae family
Source: BMC Plant Biol. 2010 Aug 16;10:176. doi: 10.1186/1471-2229-10-176 (PMC3095306; doi:10.1186/1471-2229-10-176)

Additional file 3. Comparison of divergences of genic and intergenic regions. The distances (Pi) between individual clones were calculated with the assistance of DnaSP 4.0 software [33]. The sequences were obtained in this study or downloaded from the EMBL/GenBank database. The data sets comprised rDNA sequences from *Artemisia* (2 species), *Tagetes*, *Matricaria*, *Helichrysum* and *Elachanthemum*.

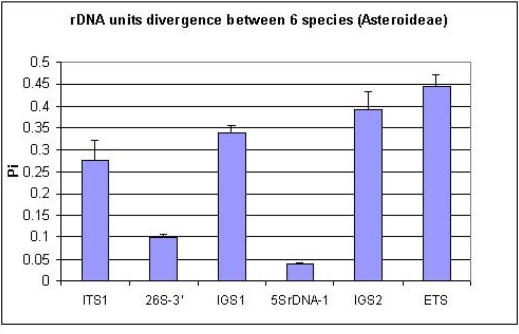

Supplement: Additional file 3 — Comparison of divergences of genic and intergenic regions. The distances (Pi) between individual clones were calculated with the assistance of DnaSP 4.0 software [33]. The sequences were obtained in this study or downloaded from the EMBL/GenBank database. The data sets comprised rDNA sequences from Artemisia (2 species), Tagetes, Matricaria, Helichrysum and Elachanthemum. [file 1471-2229-10-176-S3.PDF]
