# Supplementary material for: Repeated reunions and splits feature the highly dynamic evolution of 5S and 35S ribosomal RNA genes (rDNA) in the Asteraceae family
Source: BMC Plant Biol. 2010 Aug 16;10:176. doi: 10.1186/1471-2229-10-176 (PMC3095306; doi:10.1186/1471-2229-10-176)
Supplement: Additional file 4 — Accession number of DNA sequences used for construction of phylograms. [file 1471-2229-10-176-S4.PDF]

Additional file 4. Accession number of DNA sequences used for construction of phylograms.

		Genebank accession number	Reference
5S rDNA genic seq.			
	Nicotiana tabacum	AJ222659	[1]
	Solanum tuberosum	Y16659	[2]
	Brassica napus	X05060	[3]
	Arabidopsis thaliana	AF331003	[4]
	Secale cereale	AJ307402	[5]
	Triticum aestivum	AJ307373	[5]
	Gnaphalium luteoalbum	HM160523	This work
	Helichrysum cymosum	HM160526	This work
	Tagetes minuta	HM160525	This work
	Matricaria matricarioides	HM160524	This work
	Artemisia absinthium	EU649668	[6]
	Chrysanthemum zawadskii	EF202581	[7]
	Elachanthemum intricatum	HM160527	This work
	Funaria hygrometrica	X80212	[8]
	Neurospora crassa	K02949	[9]
Cassandra elements			
	Pisum sativum	DQ788719	[10]
	Linum usitatissiumum	DQ767972	[10]
	Sphaeropteris cooperi	AY860310	[10]
	Nephrolepsis exaltata	AY860313	[10]
	Rosa rugosa	AY860316	[10]
	Fragaria ananassa	AY860312	[10]

References

1. Fulnecek J, Matyasek R, Kovarik A, Bezdek M: **Mapping of 5-methylcytosine residues in Nicotiana tabacum 5S rRNA genes by genomic sequencing**. *Mol Gen Genet* 1998, **259**(2):133-141.

2. Volkov RA, Zanke C, Panchuk II, Hemleben V: **Molecular evolution of 5S rDNA of Solanum species (sect. Petota): application for molecular phylogeny and breeding**. *Theor Appl Genet* 2001, **103**(8):1273-1282.
3. Barciszewska MZ, Mashkova TD, Kisselev LL, Barciszewski J: **The Nucleotide-Sequences of 5s Ribosomal-Rnas from 2 Plants - Rape and White Beet**. *Nucleic Acids Res* 1987, **15**(1):363-363.
4. Cloix C, Tutois S, Yukawa Y, Mathieu O, Cuvillier C, Espagnol MC, Picard G, Tourmente S: **Analysis of the 5S RNA pool in Arabidopsis thaliana: RNAs are heterogeneous and only two of the genomic 5S loci produce mature 5S RNA**. *Genome Res* 2002, **12**(1):132-144.
5. Fulnecek J, Matyasek R, Kovarik A: **Distribution of 5-methylcytosine residues in 5S rRNA genes in Arabidopsis thaliana and Secale cereale**. *Mol Genet Genomics* 2002, **268**(4):510-517.
6. Garcia S, Lim KY, Chester M, Garnatje T, Pellicer J, Valles J, Leitch AR, Kovarik A: **Linkage of 35S and 5S rRNA genes in Artemisia (family Asteraceae): first evidence from angiosperms**. *Chromosoma* 2009, **118**(1):85-97.
7. Seo JH, Pak JH, Seo BB: **Sequence variation among tandem repeat units of 5S rRNA gene and phylogenetic relationship in four taxa of Dendranthema**. *Korean J Genetic* 2007, **29**(2):211-218.
8. Capesius I: **Analysis of the ribosomal RNA gene repeat from the moss Funaria hygrometrica**. *Plant Mol Biol* 1997, **33**(3):559-564.
9. Morzyckawroblewska E, Selker EU, Stevens JN, Metzenberg RL: **Concerted Evolution of Dispersed Neurospora-Crassa 5s Rna Genes - Pattern of Sequence Conservation between Allelic and Nonallelic Genes**. *Mol Cell Biol* 1985, **5**(1):46-51.
10. Kalendar R, Tanskanen J, Chang W, Antonius K, Sela H, Peleg O, Schulman AH: **Cassandra retrotransposons carry independently transcribed 5S RNA**. *Proc Natl Acad Sci U S A* 2008, **105**(15):5833-5838.
